# Crosslinked sulfonated polyacrylamide (Cross-PAA-SO_3_H) tethered to nano-Fe_3_O_4_ as a superior catalyst for the synthesis of 1,3-thiazoles

**DOI:** 10.1186/s13065-019-0637-0

**Published:** 2019-10-12

**Authors:** Hossein Shahbazi-Alavi, Sheida Khojasteh-Khosro, Javad Safaei-Ghomi, Maryam Tavazo

**Affiliations:** 1 Young Researchers and Elite Club, Islamic Azad University, Kashan Branch, Kashan, Iran; 2 Department of Organic Chemistry, Faculty of Chemistry, University of Kashan, Kashan, Iran

**Keywords:** Polyacrylamide, Thiazole, Nanocatalyst, Nano-Fe_3_O_4_

## Abstract

Crosslinked sulfonated polyacrylamide (Cross-PAA-SO_3_H) attached to nano-Fe_3_O_4_ as a superior catalyst has been used for the synthesis of 3-alkyl-4-phenyl-1,3-thiazole-2(3*H*)-thione derivatives through a three-component reactions of phenacyl bromide or 4-methoxyphenacyl bromide, carbon disulfide and primary amine under reflux condition in ethanol. A proper, atom-economical, straightforward one-pot multicomponent synthetic route for the synthesis of 1,3-thiazoles in good yields has been devised using crosslinked sulfonated polyacrylamide (Cross-PAA-SO_3_H) tethered to nano-Fe_3_O_4_. The catalyst has been characterized by Fourier-transform infrared spectroscopy (FT-IR), scanning electron microscope (SEM), dynamic light scattering (DLS), X-ray powder diffraction (XRD), energy-dispersive X-ray spectroscopy (EDS), thermogravimetric analysis (TGA) and vibrating-sample magnetometer (VSM).

## Introduction

**Scheme 1:**
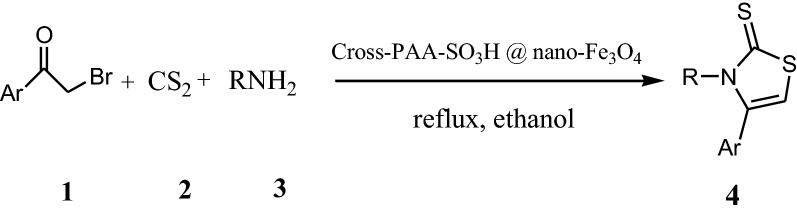
Synthesis of 1,3-thiazoles

**Scheme 2:**
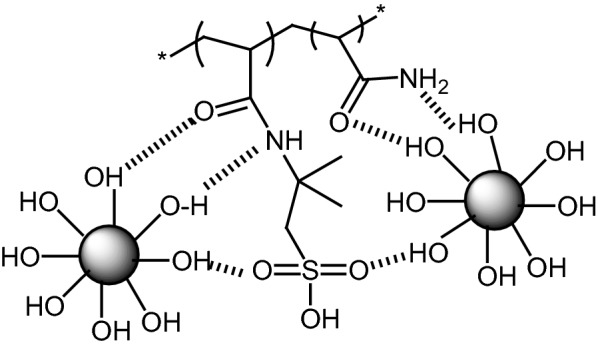
A schematic illustration for the formation of Nano Fe_3_O_4_@PAA-SO_3_H

1,3-thiazoles show anticancer [1], antimicrobial [2], anti-inflammatory [3], and anti-candida properties [4]. The synthesis of 1,3-thiazole derivatives have been developed in the presence of different catalysts including DBU [5], HClO_4_-SiO_2_ [6], Bi(SCH_2_COOH)_3_ [7], [Et_3_NH][HSO_4_] [8], Ytterbium(III) Triflate [9] 2-pyridinecarboxaldehyde oxime [10] and potassium iodide [11]. The synthetic strategies of 1,3-thiazole derivatives were recently reviewed [12]. Despite the use of these ways, there remains a need for further new procedures for the preparation of 1,3-thiazoles. The modifying crosslinked polyacrylamides make them attractive objects in chemistry and polymer science [13–15]. Sulfonated polyacrylamides have unique characteristics such as high strength, hydrophilicity, and proton conductivity [16, 17]. Recently, magnetic nanoparticles (MNPs) have been successfully utilized to immobilize enzymes, polymers, transition metal catalysts and organocatalysts [18, 19]. Different stabilizers-electrostatic (surfactants), [20] or steric (polymers) [21–23] have been proposed to overcome the aggregation of magnetite (Fe_3_O_4_). In the current study, we investigated an easy and rapid method for the synthesis of thiazole-2(3*H*)-thione through three-component reactions of phenacyl bromide or 4-methoxyphenacyl bromide, carbon disulfide and primary amine using crosslinked sulfonated polyacrylamide (Cross-PAA-SO_3_H) attached to nano-Fe_3_O_4_, as an efficient catalyst under reflux condition in ethanol (Scheme 1). A schematic representation of the catalyst is provided in Scheme 2.

## Results and discussion

### Characterization of the nanocatalyst

**Fig. 1:**
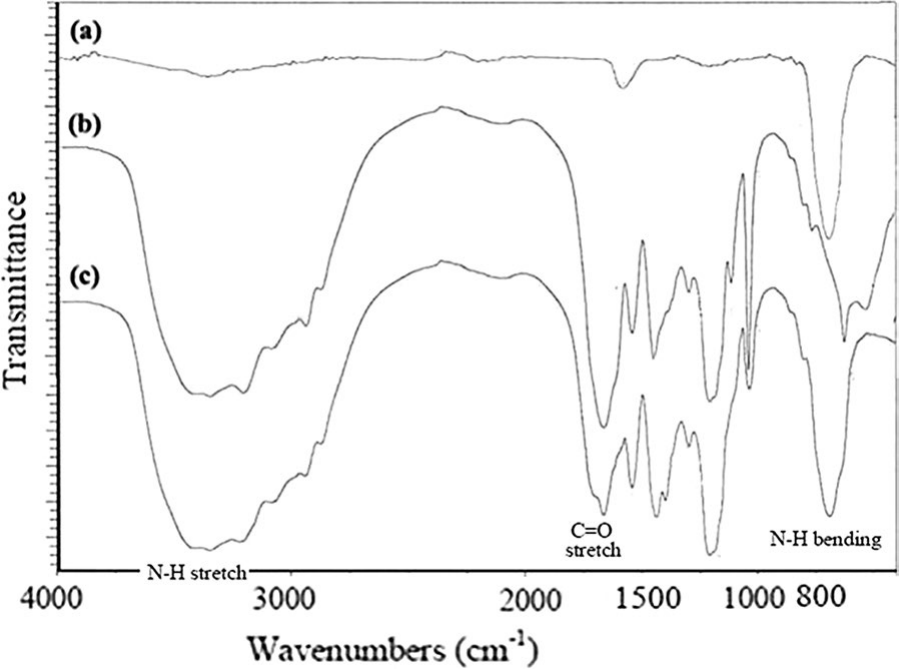
The FT-IR spectra of (a) Fe_3_O_4_ NPs, (b) Cross-PAA-SO_3_H and (c) Cross-PAA-SO_3_H@ nano-Fe_3_O_4_

In this study, we synthesized the crosslinked sulfonated polyacrylamide (Cross-PAA-SO_3_H) with simultaneous radical co-polymerization in presence of initiator and crosslinking agent. The FT-IR absorbance spectra of the dried crosslinked sulfonated polyacrylamide (poly AAM-*co*-AAMPS), Fe_3_O_4_ and Cross-PAA-SO_3_H@nano-Fe_3_O_4_ are shown in Fig. 1 (AAM is abbreviation acrylamide; AAMPS is abbreviation 2-acrylamido-2-methylpropanesulfonic acid).

**Scheme 3:**
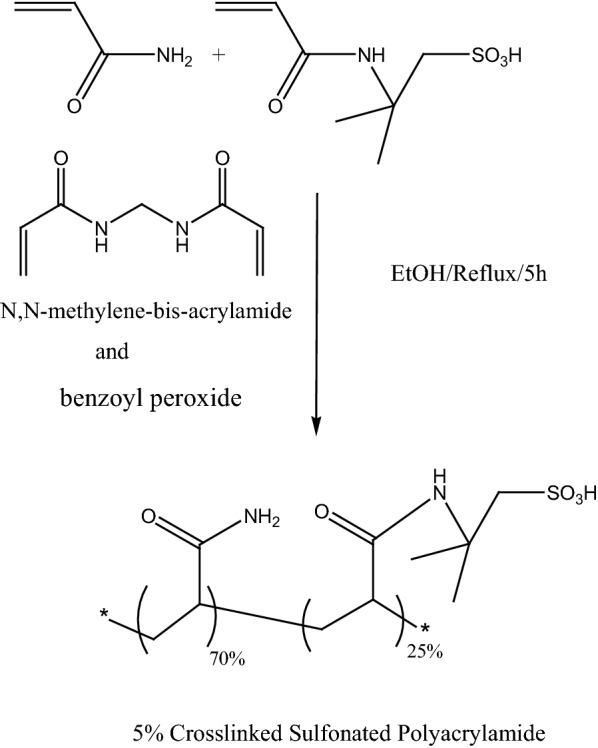
Preparation of crosslinked Sulfonated Polyacrylamide (Cross-PAA-SO_3_H)

**Fig. 2:**
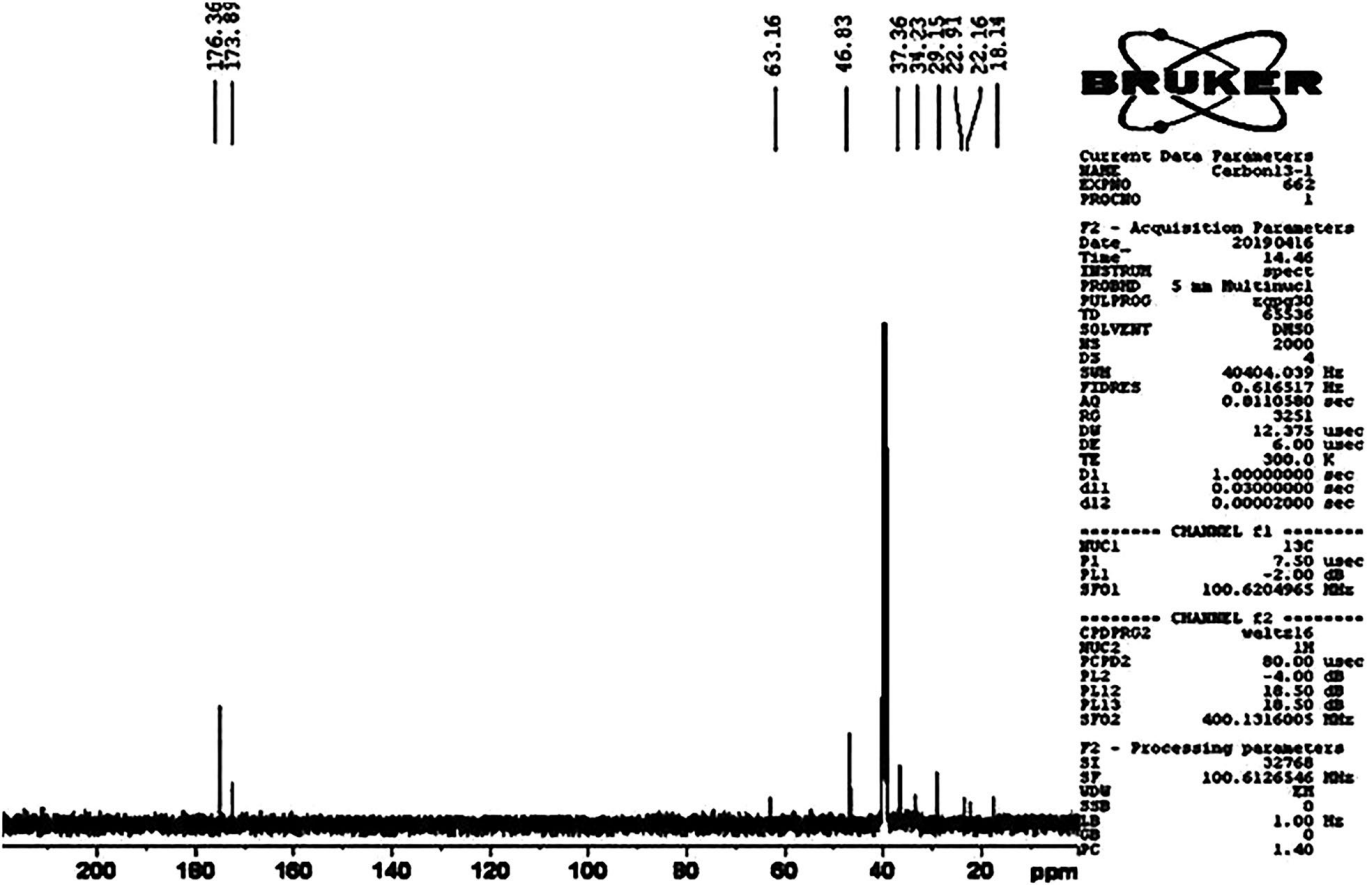
The ^13^C NMR spectra of Cross-PAA-SO_3_H

The peaks at 3100–3500 cm^−1^ are related to O–H (sulfonic acid group) and N–H (amide groups) in AAM and AAMPS. The strong band in the 1654 cm^−1^ can be ascribed to the stretching vibrations of carbonyl groups in both AAM and AAMPS. The sharp peak at 1040 cm^−1^ is related to sulfonic acid (–SO_3_H) group. The bands at 700–800 cm^−1^ and 1540 cm^−1^ are related to the bending vibration of the N–H bond (primary and secondary amide respectively). Table 1 gives the main characteristic peak assignment of the FT-IR spectra. Meanwhile, a schematic illustration of the reaction is presented in the Scheme 3. The results in Fig. 1c suggest the integration of Fe_3_O_4_ NPs and Cross-PAA-SO_3_H. The carbon nuclear magnetic resonance (^13^C NMR) of Cross-PAA-SO_3_H is displayed in Fig. 2. The peaks at 63.16 (CH_2_SO_3_H), 46.83 (CHCONH_2_), 37.36 (CNHMe_2_), 34.23 (–CCH_2_CO), 29.15 (CH_2_), 22.91, 22.16 ppm (2 CH_3_), 18.14 (CH_2_CHCONH_2_) are shown in Fig. 2. The ^13^C NMR spectrum of the Cross-PAA-SO_3_H in DMSO-*d*_*6*_ displayed two peaks at 176.36 and 173.89 ppm due to amide groups.

**Table 1:** Peak assignment of crosslinked Sulfonated Polyacrylamide (Cross-PAA-SO_3_H)

Peak position (cm^−1)^	Assignment
3100–3500	N–H stretching of NH_2_, OH stretching of (–SO_3_H)
1658	C=O stretching of CO in AAM and AAMPS
1545	N–H bending (Secondary amid band of AAMPS)
1042	Sulfonic acid (–SO_3_H) group
1175–1216	Symmetric band of SO_2_
1453	Stretching of the C–N band (amid)
700–800	N–H bending (primary amide)

**Fig. 3:**
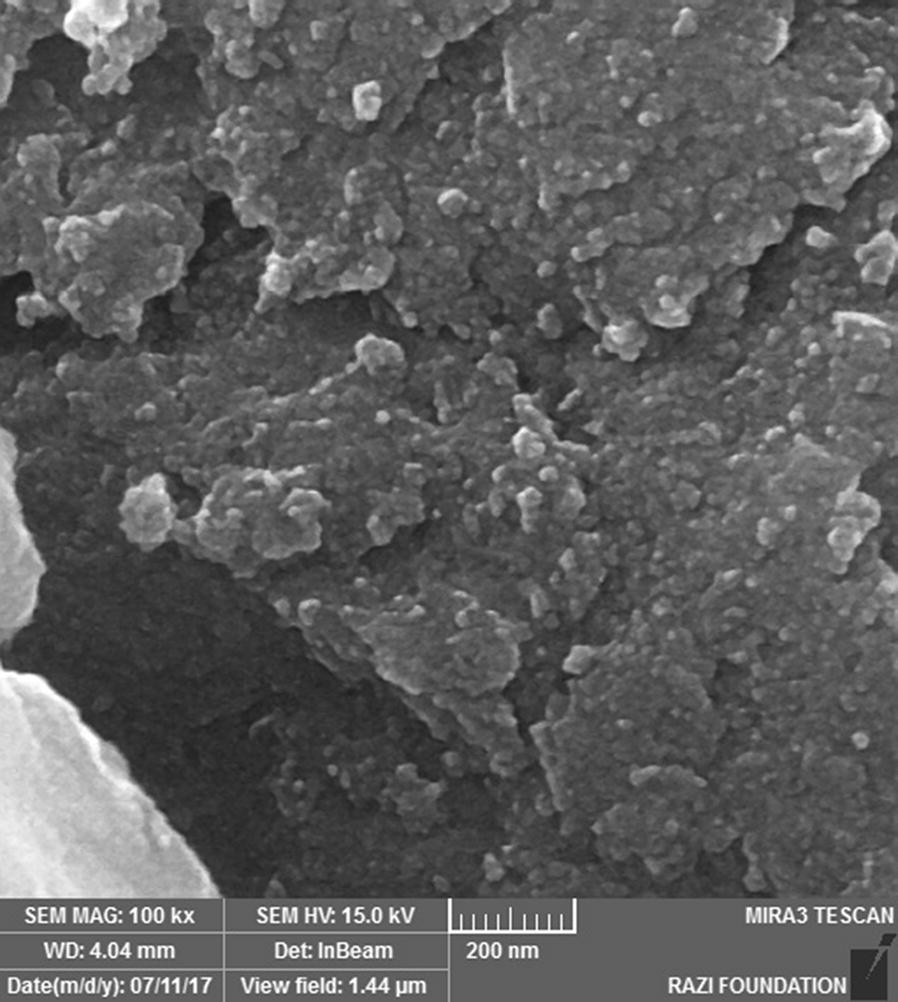
SEM image of Cross-PAA-SO_3_H@nano-Fe_3_O_4_

**Fig. 4:**
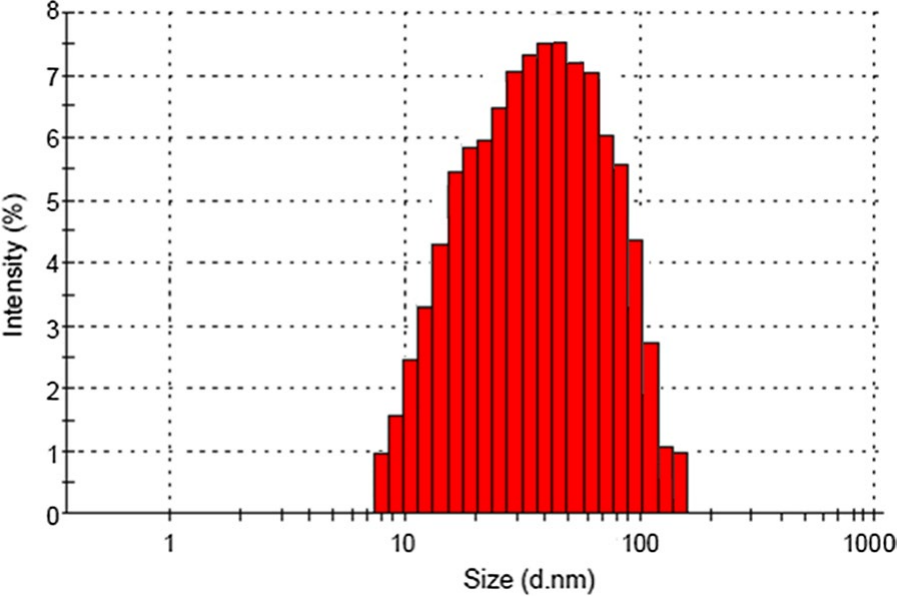
The DLS of Cross-PAA-SO_3_H@nano-Fe_3_O_4_

The morphology of Cross-PAA-SO_3_H@nano-Fe_3_O_4_ was determined by Scanning Electronic Microscopy (SEM). It is observed that the particles are strongly aggregated and glued with very large and continuous aggregates (Fig. 3). In order to investigate the size distribution of nanocatalysts [24, 25], dynamic light scattering (DLS) measurements of the nanoparticles were showed in Fig. 4. The size distribution is centered at a value of 52.4 nm. The dispersion for DLS analysis (2.5 g nanocatalyst at 50 mL ethanol) was prepared using an ultrasonic bath (60 W) for 30 min.

**Fig. 5:**
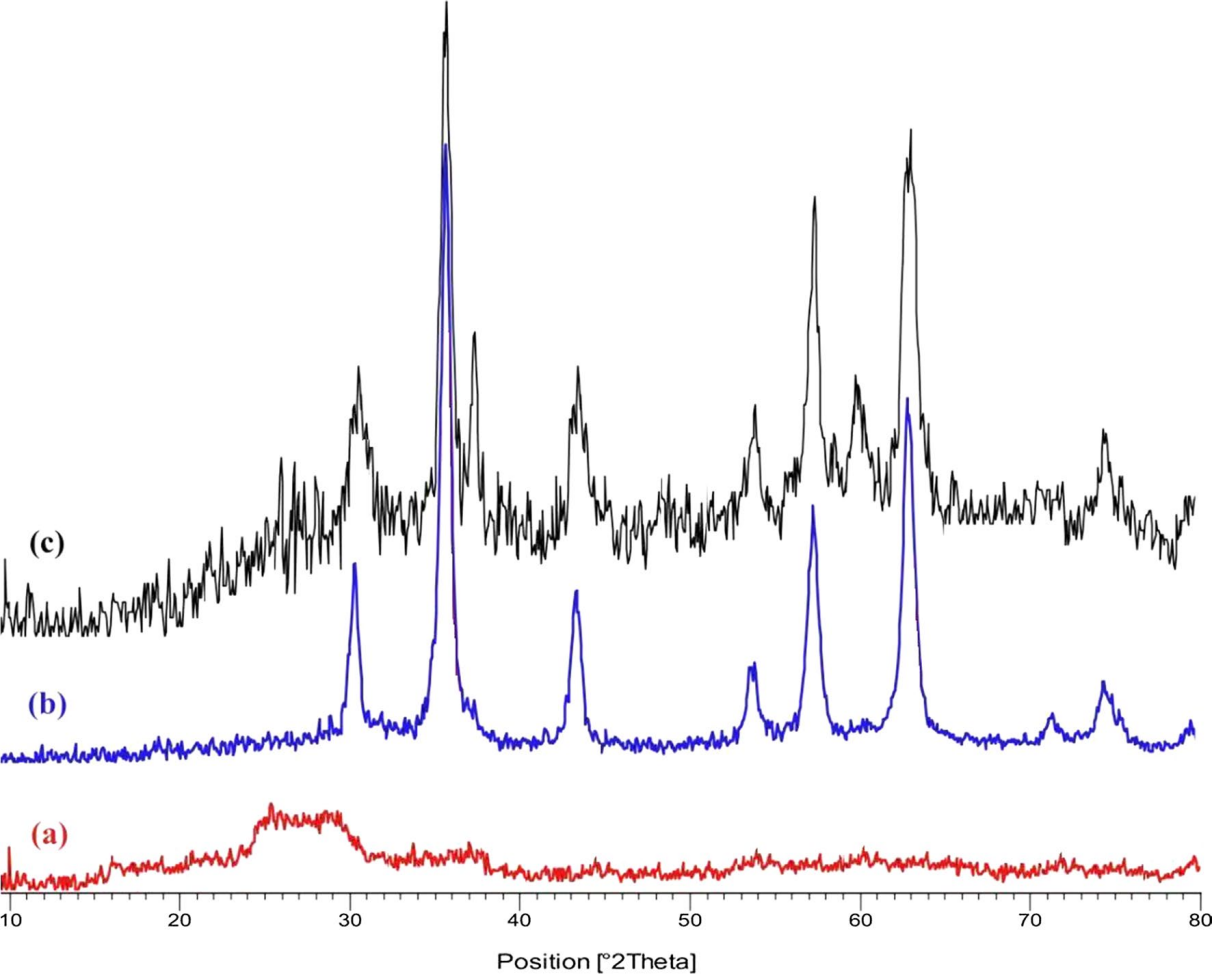
The XRD pattern of (a) Cross-PAA-SO_3_H, (b) Fe_3_O_4_ and (c) Cross-PAA-SO_3_H@nano-Fe_3_O_4_

XRD patterns of Cross-PAA-SO_3_H, Fe_3_O_4_ and Cross-PAA-SO_3_H@nano-Fe_3_O_4_ are shown in Fig. 5. The patterns for Cross-PAA-SO_3_H indicate a peak at 2θ = 28° which is the most intense peak height (Fig. 5a). All the strong peaks appeared at 2θ = 30.08°, 35.40°, 43.17°, 53.59°, 57.20°, 62.86°, and 74.02° can be easily indexed to nano-Fe_3_O_4_ (Fig. 5b). The pattern agrees well with the reported pattern for Fe_3_O_4_ (JCPDS No. 75-1609). The particle size diameter (D) of the nanoparticles has been calculated by the Debye–Scherrer equation (D = Kλ/β cosθ), where β FWHM (full-width at half-maximum or half-width) is in radian and θ is the position of the maximum of the diffraction peak. K is the so-called shape factor, which usually takes a value of about 0.9, and λ is the X-ray wavelength (1.5406 Å for CuKα). The crystallite size of Cross-PAA-SO_3_H@nano-Fe_3_O_4_ was calculated by the Debye–Scherer equation is about 48–52 nm. The weaker diffraction lines of Cross-PAA-SO_3_H@nano-Fe_3_O_4_ (Fig. 5c) compared with Fe_3_O_4_ nanoparticles indicate that the Fe_3_O_4_ nanoparticles were covered by amorphous polymer.

**Fig. 6:**
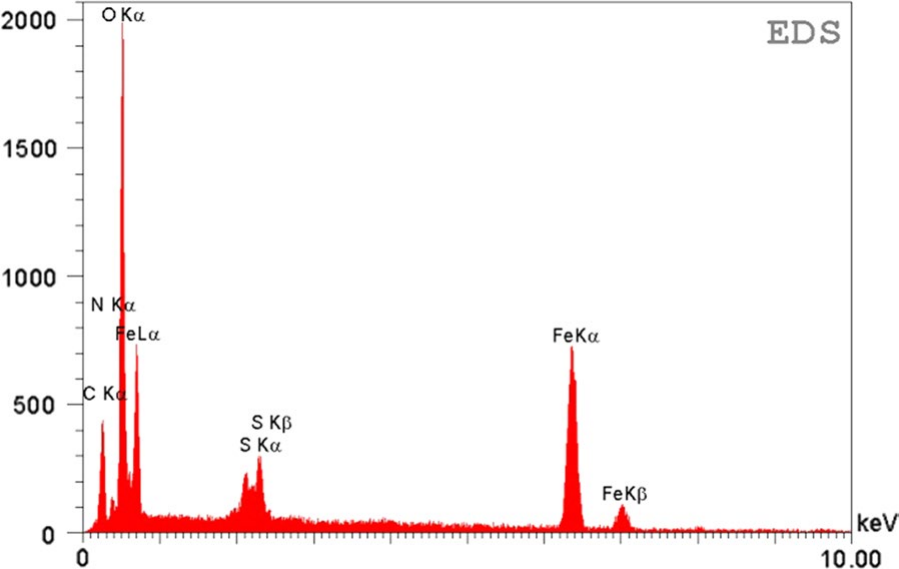
EDS spectrum of Cross-PAA-SO_3_H@nano-Fe_3_O_4_

An EDS (energy dispersive X-ray) spectrum of Cross-PAA-SO_3_H@nano-Fe_3_O_4_ (Fig. 6) exhibits that the elemental compositions are carbon, oxygen, sulfur, iron and nitrogen.

**Fig. 7:**
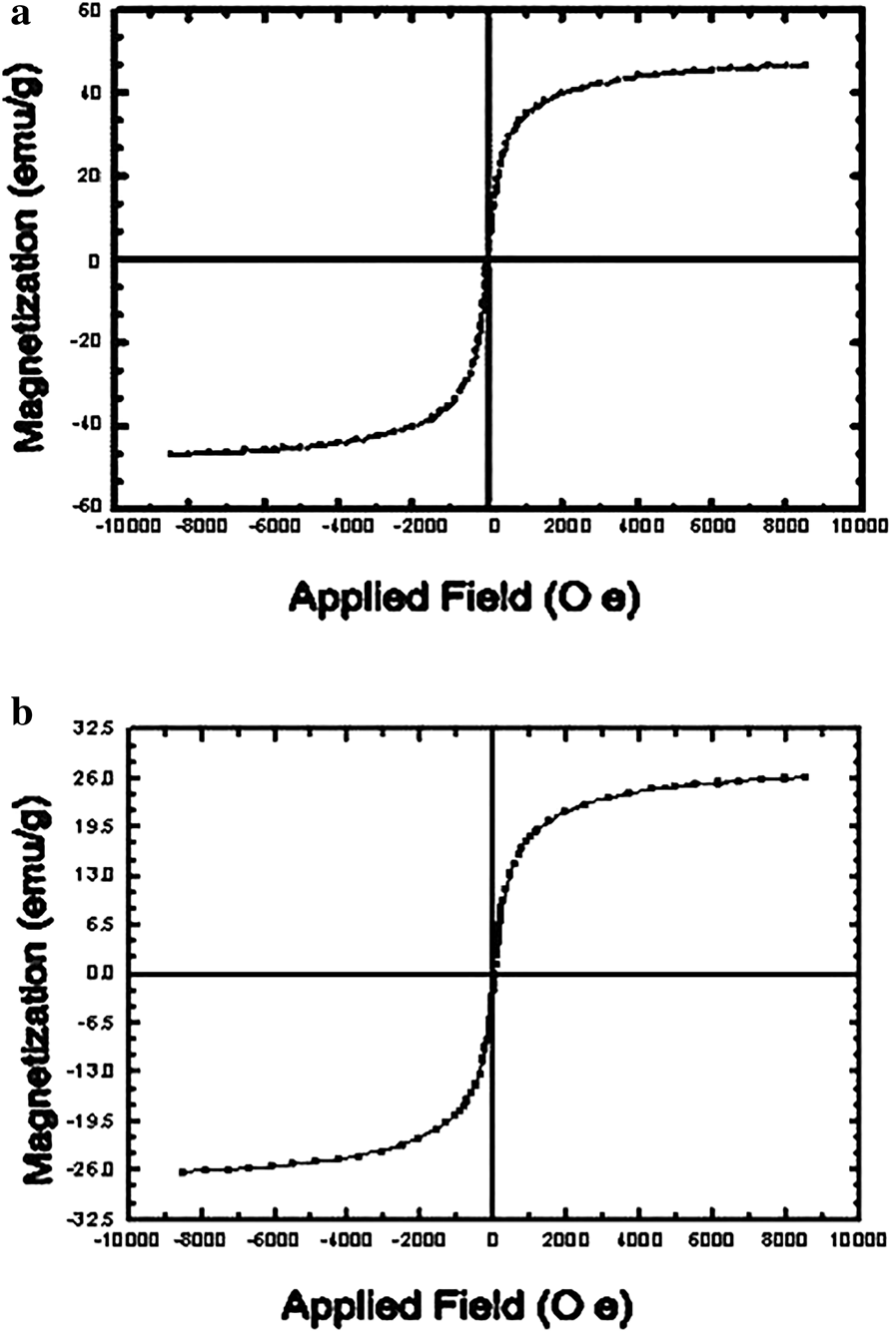
The VSM curve of: **a** nano-Fe_3_O_4_ and **b** Cross-PAA-SO_3_H@nano-Fe_3_O_4_

The magnetic attributes of nano-Fe_3_O_4_ and Cross-PAA-SO_3_H@nano-Fe_3_O_4_ were given with the help of a vibrating sample magnetometer (VSM) (Fig. 7). The amount of saturation-magnetization for nano-Fe_3_O_4_ and Cross-PAA-SO_3_H@nano-Fe_3_O_4_ is 47.2 emu/g and 26.8 emu/g.

**Fig. 8:**
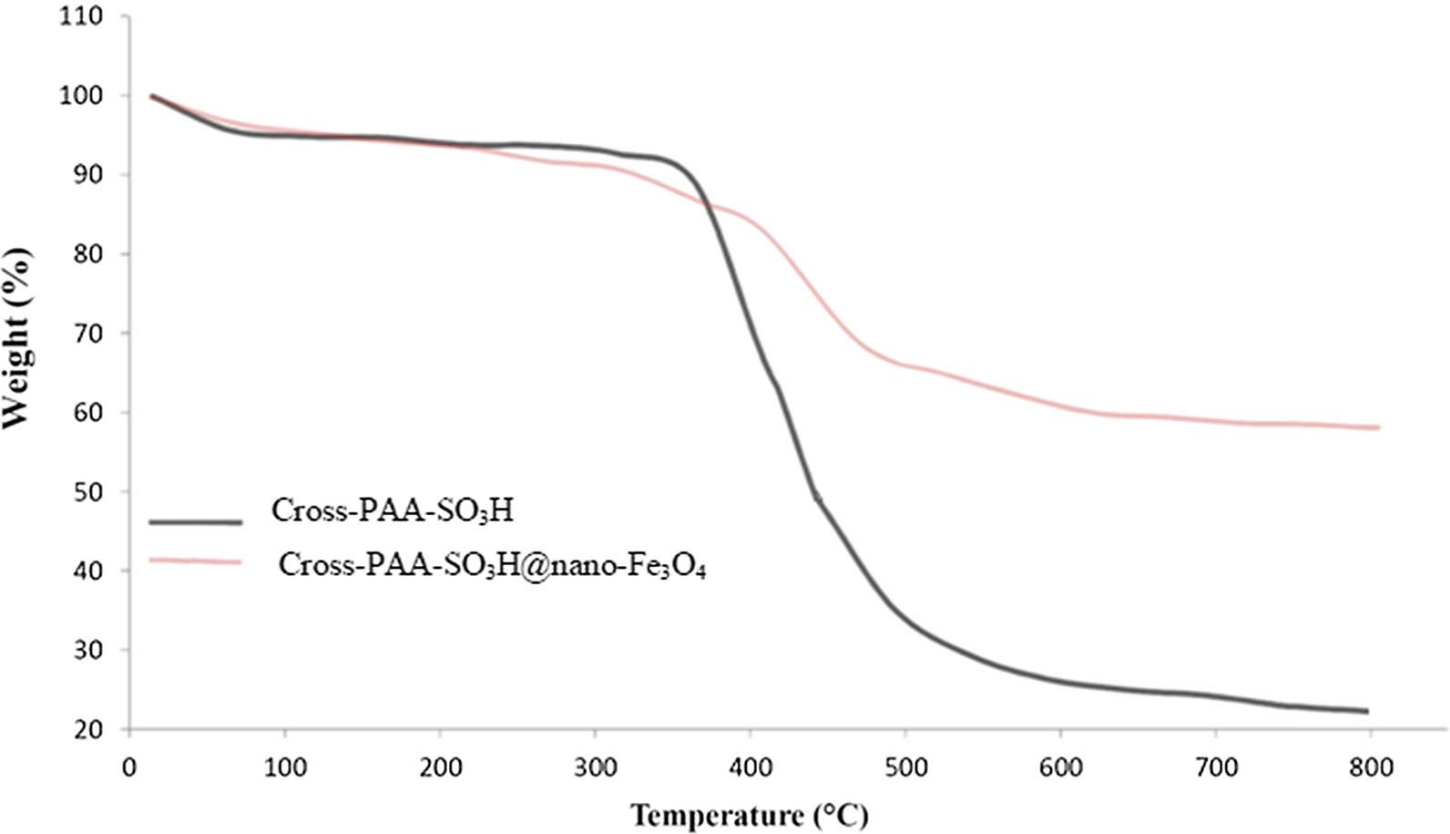
TGA curve of Cross-PAA-SO_3_H and Cross-PAA-SO_3_H@nano-Fe_3_O_4_

Thermogravimetric analysis (TGA) evaluates the thermal stability of the Cross-PAA-SO_3_H and Cross-PAA-SO_3_H@nano-Fe_3_O_4_. The curve displays a weight loss about 37.5% for Cross-PAA-SO_3_H@nano-Fe_3_O_4_ from 240 to 550 °C, resulting from the destruction of organic spacer attaching to the nanoparticles. Hence; the nanocatalyst was stable up to 240 °C, confirming that it could be stably utilized in organic reactions at temperatures between the ranges of 80–160 °C (Fig. 8).

### Catalytic behaviors of Cross-PAA-SO_3_H@nano-Fe_3_O_4_ for the synthesis of 1,3-thiazoles

Initially, we had optimized conditions for the synthesis of 3-alkyl-4-phenyl-1,3-thiazole-2(3*H*)-thione derivatives by the reaction of phenacyl bromide, carbon disulfide and benzyl amine as a model reaction. The model reactions were performed by CAN, NaHSO_4_, InCl_3_, ZrO_2_, *p*-TSA, nano-Fe_3_O_4_, Cross-PAA-SO_3_H and Cross-PAA-SO_3_H@nano-Fe_3_O_4_. The reactions were tested using diverse solvents including ethanol, acetonitrile, water or dimethylformamide. The best results were gained in EtOH and we found that the reaction gave convincing results in the presence of cross-PAA-SO_3_H@nano-Fe_3_O_4_ (7 mg) under reflux conditions (Tables 2). However, the activity of catalysts is determined by the acid–base properties, surface area, the distribution of sites and the polarity of the surface sites [26, 27]. We studied the feasibility of the reaction by selecting some representative substrates (Table 3). To investigate the extent this catalytic process, phenacyl bromide or 4-methoxyphenacyl bromide, carbon disulfide and primary amine were elected as substrates. Seeking of the reaction scope demonstrated that various primary amines can be utilized in this method (Table 3).

**Table 2:** Optimization of reaction conditions

Entry	Solvent (reflux)	Catalyst	Time (min)	Yield (%)^a^
1	EtOH	–	500	39
2	EtOH	CAN (7 mol%)	250	53
3	EtOH	NaHSO_4_ (5 mol%)	300	45
4	EtOH	InCl_3_ (4 mol%)	200	56
5	EtOH	ZrO_2_ (6 mol%)	250	60
6	EtOH	*p*-TSA (3 mol%)	200	64
7	EtOH	Nano-Fe_3_O_4_ (10 mg)	200	52
8	EtOH	Cross-PAA-SO_3_H (10 mg)	150	56
9	EtOH	nano-Fe_3_O_4_ (5 mg) + Cross-PAA-SO_3_H (5 mg)	150	60
10	H_2_O	Cross-PAA-SO_3_H@nano-Fe_3_O_4_ (7 mg)	150	77
11	DMF	Cross-PAA-SO_3_H@nano-Fe_3_O_4_ (7 mg)	150	82
12	CH_3_CN	Cross-PAA-SO_3_H@nano-Fe_3_O_4_ (7 mg)	150	89
13	EtOH	Cross-PAA-SO_3_H@nano-Fe_3_O_4_ (5 mg)	150	92
14	EtOH	Cross-PAA-SO_3_H@nano-Fe_3_O_4_ (7 mg)	150	94
15	EtOH	Cross-PAA-SO_3_H@nano-Fe_3_O_4_ (9 mg)	150	94

Phenacyl bromide (1 mmol), carbon disulfide (1 mmol) and benzyl amine (1 mmol)

^a^Isolated yield

**Table 3:**
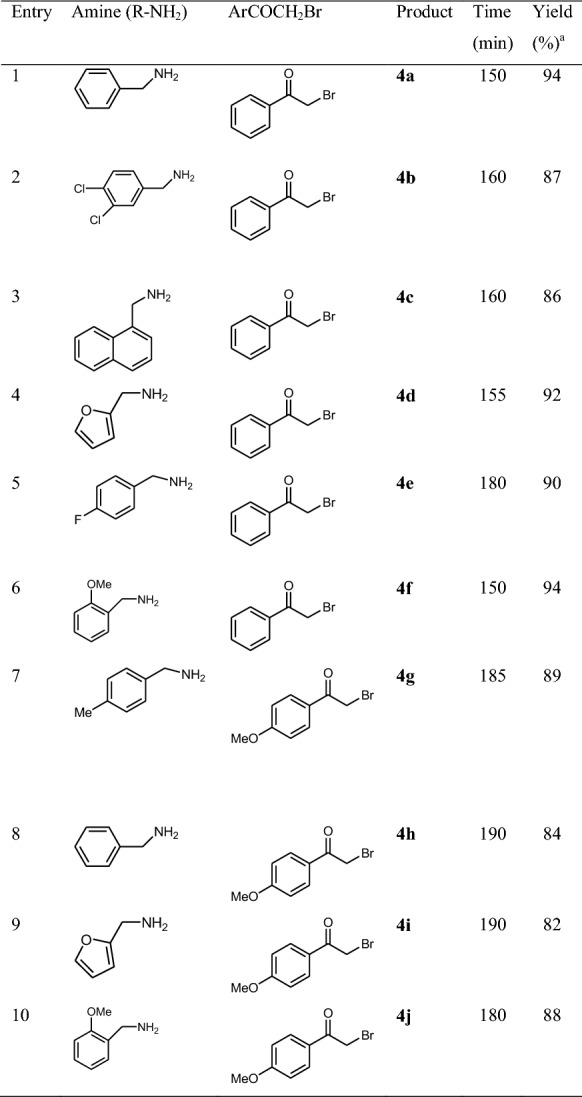
Synthesis of thiazoles using Cross-PAA-SO_3_H@nano-Fe_3_O_4_

^a^Isolated yield

**Scheme 4:**
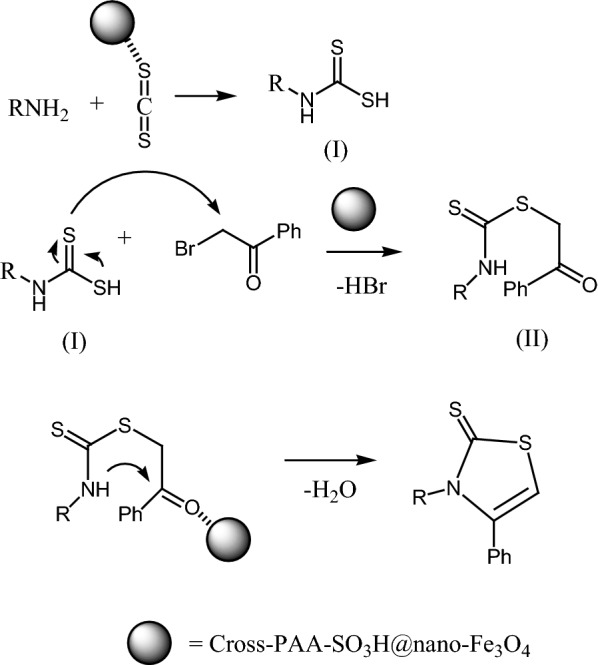
Proposed reaction pathway for the synthesis of 1,3-thiazoles

Scheme 4 displays a proposed mechanism for this reaction in the presence of cross-PAA-SO_3_H@nano-Fe_3_O_4_ as catalyst. Initially the nucleophilic attack by amines on a carbon disulfide generates intermediate (I), The next step involves nucleophilic attack of intermediate (I) on the methylene carbon of phenacyl bromide, leading to intermediate (II), and then ring closure by intramolecular attack of nitrogen at the carbonyl carbon to afford the 3-alkyl-4-phenyl-1,3- thiazole-2(3*H*)-thione derivatives **4**. In this mechanism the surface atoms of cross-PAA-SO_3_H@nano-Fe_3_O_4_ activate the C=S and C=O groups for better reaction with nucleophiles.

The reusability of Cross-PAA-SO_3_H@nano-Fe_3_O_4_ was studied for the reaction of phenacyl bromide, carbon disulfide and benzyl amine and it was found that product yields reduced to a small extent on each reuse (run 1, 94%; run 2, 94%; run 3, 93%; run 4, 93%; run 5, 92%; run 6, 92%;). After completion of the reaction, the nanocatalyst was separated by an external magnet. The catalyst was washed four times with ethanol and dried at room temperature for 18 h. The possibility of recycling of the catalyst is an important process from different aspects such as environmental concerns, and commercial applicable processes.

To study the applicability of this method in larger scale synthesis, we performed selected reactions at 10 mmol scale. As can be seen, the reactions at large scale gave the product with a gradual decreasing of reaction yield (Table 4).

**Table 4:** The large-scale synthesis of some 1,3-thiazoles using cross-PAA-SO_3_H@nano-Fe_3_O_4_

Entry	Product	Time (min)	Yield (%)^a^
1	**4a**	200	90
2	**4e**	200	84
3	**4g**	250	82
4	**4i**	250	75
5	**4j**	250	78

To compare the efficiency of Nano Fe_3_O_4_@ PAA-SO_3_H with the reported catalysts for the synthesis of 1,3-thiazoles, we have tabulated the results in Table 5. As Table 5 indicates, nano Fe_3_O_4_@ PAA-SO_3_H is superior with respect to the reported catalysts in terms of reaction time, yield and conditions. As expected, the increased surface area due to small particle size increased reactivity of catalyst. This factor is responsible for the accessibility of the substrate molecules on the catalyst surface.

**Table 5:** Comparison of catalytic activity of nano Fe_3_O_4_@ PAA-SO_3_H with other reported catalysts for the synthesis 1,3-thiazoles

Entry	Catalyst (condition)	Time (min)	Yield^a^, %	[Refs]
1	Bi(SCH_2_COOH)_3_ (15 mol%, 70 °C)	180	80	[7]
2	Yb(OTf)_3_ (15 mol%)	240	60	[9]
2	2-pyridinecarboxaldehyde oxime (20 mol%, DMF)	400	85	[10]
3	potassium iodide (10 mol%, CH_3_OH)	400	80	[11]
4	Nano Fe_3_O_4_@ PAA-SO_3_H (7 mg, EtOH (under reflux condition)	150	94	This work

^a^Isolated yield

## Conclusions

In conclusion, we have reported an efficient way for the synthesis of 3-alkyl-4-phenyl-1,3-thiazole-2(3*H*)-thione derivatives using cross-PAA-SO_3_H@nano-Fe_3_O_4_ under reflux condition in ethanol. The method offers several advantages including easy availability, high yields, shorter reaction times, reusability of the catalyst and low catalyst loading. The present catalytic procedure is extensible to a wide diversity of substrates for the synthesis of a variety-oriented library of thiazoles.

## Experimental section

### Chemicals and apparatus

NMR spectra were obtained on a Bruker spectrometer with CDCl_3_ as solvent and TMS as an internal standard. Chemical shifts (δ) are given in ppm and coupling constants (J) are given in Hz. FT-IR spectra were recorded with KBr pellets by a Magna-IR, spectrometer 550 Nicolet. CHN compositions were measured by Carlo ERBA Model EA 1108 analyzer. Powder X-ray diffraction (XRD) was carried out on a Philips diffractometer of X’pert Company with monochromatized Cu Kα radiation (λ = 1.5406 Å). Microscopic morphology of products was visualized by SEM (MIRA3). The thermogravimetric analysis (TGA) curves are recorded using a V5.1A DUPONT 2000. The mass spectra were recorded on a Joel D-30 instrument at an ionization potential of 70 eV. The magnetic property of magnetite nanoparticle has been measured with a vibrating sample magnetometer (VSM) (Meghnatis Daghigh Kavir Co.; Kashan Kavir; Iran) at room temperature.

#### Preparation of crosslinked sulfonated polyacrylamide (Cross-PAA-SO_3_H)

In a round-bottom flask (200 mL) equipped with magnetic stirrer and condenser, 5 g of acrylamide (AAM) (70 mmol) and 5.17 g of 2-acrylamido-2-methylpropanesulfonic acid (25 mmol) (AAMPS), [approximately AAM/AAMMPS (3/1)] and 0.77 g of *N*,*N*-methylene-bis-acrylamide (NNMBA) (5 mmol) as crosslinking agent and benzoyl peroxide as initiator were added to 80 mL EtOH under reflux condition for 5 h. After completion of reaction, the white precipitate was formed, filtered, washed and dried in vacuum oven in 70 °C for 12 h. The weight of polymer was 10.1 gr with the yield of 91.8%. Cross-PAA-SO_3_H was characterized with infrared spectroscopy and back titration acid–base to confirm sulfonation and determine accurate sulfonation levels. Acidic capacity of this catalyst was estimated 1.1 mmol/g.

#### Preparation of crosslinked sulfonated polyacrylamide@nano-Fe_3_O_4_

1 gr of synthesized polymers were poured in 100 mL round bottom flask under stirring at room-temperature, then 50 mL HCl (0.4 M) was added to it. Our target molecules was synthesized by magnetic nanocatalyst with mass ratio polymer/nano-Fe_3_O_4_ = 2/1. So, 0.43 g (2.1 mol) FeCl_2_·4H_2_O and 1.17 g (2 × 2.1) FeCl_3_·6 H_2_O were added and the mixture was stirred until dissolved completely (flask1). In another 500 ml round-bottom flask no 2, 400 mL aqueous solution of NH_3_ (0.7 M) was poured under argon gas. Then flask 1 was added to flask 2 immediately. Nanocatalyst was filtered and washed with water (2 × 25 mL) and dried in oven on 50 °C.

#### General procedure for the synthesis of 1,3-thiazoles

A mixture of primary amine (1.0 mmol) and carbon disulfide (1.0 mmol) in ethanol (8 mL) was stirred for 5 min and then phenacyl bromide or 4-methoxyphenacyl bromide (1.0 mmol) and Cross-PAA-SO_3_H attached to nano-Fe_3_O_4_ (7 mg) were added, and the mixture was stirred for the appropriate times. The reaction was monitored by TLC (*n*-hexane/ethyl acetate 8:2). After completion of the reaction, the nanocatalyst was easily separated using an external magnet. The solvent was evaporated and the solid obtained washed with EtOH to get pure product. The characterization data of the compounds are given below and in Additional file 1.

##### 3-Benzyl-4-phenyl-1,3-thiazole-2(3H)-thione (**4a**)

Colorless viscous oil; FT-IR (KBr): $\bar{\nu }$ = 3102, 3005, 1602, 1479, 1202 cm^−1^; ^1^H NMR (250 MHz, CDCl_3_): *δ* 4.90 (s, 2H, CH_2_), 6.03 (s, 1H, CH of alkene), 6.95–7.36 (m, 10H, CH, ArH). ^13^C NMR (62.5 MHz, CDCl_3_): *δ* 47.24, 98.85, 127.06, 127.42, 128.52, 128.55, 129.08, 133.32, 137.45, 154.85, 178.37, 197.18. MS (EI, 70 eV): *m*/*z* (%) = 283 (5), 267 (68), 181 (7), 91 (100), 77 (4), 65 (12), 45 (4). Anal. Calcd. for C_16_H_13_NS_2_ (283): C, 67.81; H, 4.62; N, 4.94. Found: C, 67.70; H, 4.52; N, 4.73%.

##### 3-(3,4-dichlorobenzyl)-4-phenyl-1,3-thiazole-2(3H)-thione (**4b**)

Colorless viscous oil; FT-IR (KBr): $\bar{\nu }$ = 3152, 3004, 1628, 1603, 1477, 1302, 1104 cm^−1^. ^1^H NMR (250 MHz, CDCl_3_): *δ* 4.83 (s, 2H, CH_2_), 6.05 (CH of alkene), 6.75–7.97 (m, 8H, CH of ArH). ^13^C NMR (62.5 MHz, CDCl_3_): *δ* 46.07, 99.25, 126.72, 128.65, 128.74, 129.35, 129.66, 133.54, 130.50, 135.38, 136.62, 137.21, 172.70, 194.15. Anal. Calcd. for C_16_H_11_Cl_2_NS_2_ (350): C, 54.55; H, 3.15; N, 3.98. Found: C, 54.36; H, 3.05; N, 3.84%.

##### 3-(2-Naphthyl methyl)-4-phenyl-1,3-thiazole-2(3H)-thione (**4c**)

Colorless viscous oil; FT-IR (KBr): $\bar{\nu }$ = 3102, 3009, 1652, 1605, 1479, 1204 cm^−1^. ^1^H NMR (250 MHz, CDCl_3_): *δ* 3.95 (s, 2H, CH_2_), 6.12 (s, 1H, CH of alkene), 6.92–7.97 (m, 12H, CH of ArH). ^13^C NMR (62.5 MHz, CDCl_3_): *δ* 45.35, 99.05, 123.77, 125.32, 125.84, 126.34, 128.06, 128.68, 128.75, 133.54, 122.52, 129.28, 131.50, 135.08, 172.44, 194.16. Anal. Calcd. for C_20_H_15_NS_2_ (333): C, 72.03; H, 4.53; N, 4.20. Found: C, 72.05; H, 4.40; N, 4.15%.

##### 3-(2-Furyl methyl)-4-phenyl-1,3-thiazole-2(3H)-thione (**4d**)

Colorless viscous oil; FT-IR (KBr): $\bar{\nu }$ = 3105, 3002, 1653, 1607, 1474, 1202 cm^−1^. ^1^H NMR (250 MHz, CDCl_3_): *δ* 4.84 (s, 2H, CH_2_), 6.10 (s, 1H, CH of alkene), 6.22 (1H, CH of furan), 7.25–8.05 (m, 7H, CH of ArH and CH of furan). ^13^C NMR (62.5 MHz, CDCl_3_): *δ* 44.25, 98.32, 109.52, 110.83, 127.08, 128.76, 129.58, 142.12, 144.54, 147.92, 155.44, 192.18. Anal. Calcd. for C_14_H_11_NOS_2_ (273): C, 61.51; H, 4.06; N, 5.12. Found: C, 61.46; H, 4.04; N, 5.09%.

##### 3-(4-Fluorobenzyl)-4-phenyl-1,3-thiazole-2(3H)-thione (**4e**)

Colorless viscous oil; FT-IR (KBr): $\bar{\nu }$ = 3153, 3005, 1628, 1604, 1473, 1302, 1108 cm^−1^. ^1^H NMR (250 MHz, CDCl_3_): *δ* 4.85 (s, 2H, CH_2_), 6.05 (s, 1H, CH of alkene), 6.85 (d, 2H, *J *= 6.8 Hz, CH arom), 7.02–7.59 (m, 5H, CH of ArH), 7.98 (d, 2H, *J *= 7.5 Hz, CH of ArH).^13^C NMR (62.5 MHz, CDCl_3_): *δ* 46.45, 99.08, 114.53, 128.67, 128.78, 133.51, 129.05, 135.46, 137.57, 153.28, 159.50, 194.19. Anal. Calcd. for C_16_H_12_FNS_2_ (301): C, 63.76; H, 4.01; N, 4.65. Found: C, 63.60; H, 4.04; N, 4.42%.

##### 3-(2-Methoxybenzyl)-4-phenyl-1,3-thiazole-2(3H)-thione (**4f**)

Colorless viscous oil; FT-IR (KBr): $\bar{\nu }$ = 3150, 3000, 1650, 1600, 1470, 1200, 1100 cm^−1^. ^1^H NMR (250 MHz, CDCl_3_): *δ* 3.62 (s, 3H, OCH_3_), 4.90 (s, 2H, CH_2_), 6.03 (s, 1H, CH of alkene), 6.71–7.98 (m, 9H, CH, ArH).^13^C NMR (62.5 MHz, CDCl_3_): *δ* 42.56, 55.05, 98.47, 110.05, 120.53, 128.38, 128.46, 128.55, 128.78, 129.05, 133.54, 127.12, 135.45, 156.35, 194.14. Anal. Calcd. for C_17_H_15_NOS_2_ (313): C, 65.14; H, 4.82; N, 4.47. Found: C, 65.03; H, 4.74; N, 4.35%.

##### 3-(4-Methylbenzyl)-4-(4-methoxyphenyl)-1,3-thiazole-2(3H)-thione (**4g**)

Colorless viscous oil; FT-IR (KBr): $\bar{\nu }$ = 3156, 3008, 1648, 1612, 1475, 1206, 1108 cm^−1^. ^1^H NMR (250 MHz, CDCl_3_): *δ* 2.23 (s, 3H, CH_3_), 3.86 (s, 3H, OCH_3_), 4.95 (s, 2H, CH_2_), 5.98 (s, 1H, CH of alkene), 6.82–7.35 (m, 8H, CH of ArH).^13^C NMR (62.5 MHz, CDCl_3_): *δ* 21.35, 48.54, 55.95, 98.68, 115.38, 123.42, 125.64, 130.65, 131.25, 132.59, 139.25, 160.20, 174.25, 183.56. Anal. Calcd. for C_18_H_17_NOS_2_ (327): C, 66.02; H, 5.23; N, 4.28;. Found: C, 65.90; H, 5.14; N, 4.12%.

##### 3-benzyl-4-(4-methoxyphenyl)-1,3-thiazole-2(3H)-thione (**4h**)

Colorless viscous oil; FT-IR (KBr): $\bar{\nu }$ = 3157, 3012, 1645, 1616, 1478, 1209, 1107 cm^−1^. ^1^H NMR (250 MHz, CDCl_3_): *δ* 3.89 (s, 3H, OCH_3_), 5.25 (s, 2H, CH_2_), 6.28 (s, 1H, CH of alkene), 6.85–7.39 (m, 9H, CH of ArH).^13^C NMR (62.5 MHz, CDCl_3_): *δ* 48.50, 55.37, 99.86, 110.55, 114.54, 122.54, 128.38, 129.54, 132.86, 137.54, 145.68, 160.85, 185.36. MS (EI, 70 eV): *m*/*z* (%) = 313 (M). Anal. Calcd. for C_17_H_15_NOS_2_ (313): C, 65.14; H, 4.82; N, 4.47; Found: C, 65.02; H, 4.56; N, 4.34; %.

##### 3-(2-Furyl methyl)-4-(4-methoxyphenyl)-1,3-thiazole-2(3H)-thione (**4i**)

Colorless viscous oil; FT-IR (KBr): $\bar{\nu }$ = 3144, 3012, 1658, 1615, 1478, 1209, 1112 cm^−1^. ^1^H NMR (250 MHz, CDCl_3_): *δ* 3.88 (s, 3H, OCH_3_), 4.82 (s, 2H, CH_2_), 5.98 (2H, CH of furan), 6.20 (s, 1H, CH of alkene), 6.75–7.42 (m, 5H, CH of furan and CH of ArH). ^13^C NMR (62.5 MHz, CDCl_3_): *δ* 41.35, 55.34, 98.36, 108.35, 110.35, 118.35, 122.54, 130.22, 138.54, 142.35, 150.65, 161.25, 178.25. Anal. Calcd. for C_15_H_13_NO_2_S_2_ (303): C, 59.38; H, 4.32; N, 4.62; Found: C, 59.15; H, 4.14; N, 4.42. %.

##### 3-(2-methoxybenzyl)-4-(4-methoxyphenyl)-1,3-thiazole-2(3H)-thione (**4j**)

Colorless viscous oil; FT-IR (KBr): $\bar{\nu }$ = 3142, 3010, 1654, 1611, 1472, 1205, 1116 cm^−1^. ^1^H NMR (250 MHz, CDCl_3_): *δ* 3.68 (s, 3H, OCH_3_), 3.84 (s, 3H, OCH_3_), 4.89 (s, 2H, CH_2_), 6.05 (s, 1H, CH of alkene), 6.72–7.53 (m, 8H, CH of ArH). ^13^C NMR (62.5 MHz, CDCl_3_): *δ* 43.54, 56.45, 56.48, 98.45, 110.25, 115.28, 120.54, 122.54, 125.85, 125.64, 128.54, 130.42, 138.20, 158.64, 160.24, 172.54. Anal. Calcd. for C_18_H_17_NO_2_S_2_ (343): C, 62.94; H, 4.99; N, 4.08; Found: C, 62.72; H, 4.70; N, 3.91. %.

## Supplementary information

**Additional file 1.** The spectral data of products are described in the additional file 1.

## Data Availability

All data generated or analysed during this study are included in this published article [and its additional information files].

## Abbreviations

Cross-PAA-SO_3_H: crosslinked sulfonated polyacrylamide
FT-IR: Fourier-transform infrared spectroscopy
SEM: scanning electron microscope
XRD: X-ray powder diffraction
EDS: energy-dispersive X-ray spectroscopy
TGA: thermogravimetric analysis
VSM: vibrating-sample magnetometer
AAM: acrylamide
AAMPS: 2-acrylamido-2-methylpropanesulfonic acid
DLS: dynamic light scattering
