# Supplementary material for: Crosslinked sulfonated polyacrylamide (Cross-PAA-SO3H) tethered to nano-Fe3O4 as a superior catalyst for the synthesis of 1,3-thiazoles
Source: BMC Chem. 2019 Oct 12;13(1):120. doi: 10.1186/s13065-019-0637-0 (PMC6790011; doi:10.1186/s13065-019-0637-0)

**Crosslinked sulfonated polyacrylamide (Cross-PAA-SO_3_H) tethered to nano-Fe_3_O_4_ as a superior catalyst for the synthesis of 1,3-thiazoles**

Hossein Shahbazi-Alavi*,^a^ Sheida Khojasteh-Khosro,^b^ Javad Safaei-Ghomi,^b^ Maryam Tavazo,^b^

*^a^ Young Researchers and Elite Club, Kashan Branch, Islamic Azad University, Kashan, Iran ^b^Department of Organic Chemistry, Faculty of Chemistry, University of Kashan,*

*Corresponding author. E-mail addresses: hossien_shahbazi@yahoo.com, Fax: +98-31-55427233; Tel.: +98-31-55427233*

*3-Benzyl-4-phenyl-1,3-thiazole-2(3H)-thione* (**4a**):

Colorless viscous oil; FT-IR (KBr): = 3102, 3005, 1602, 1479, 1202 cm^-1^; ^1^H NMR (250 MHz, CDCl_3_*)*: *δ* 4.90 (s, 2H, CH_2_*)*, 6.03 (s, 1H, CH of alkene), 6.95–7.36 (m, 10H, CH, ArH). ^13^C NMR (62.5 MHz, CDCl_3_*)*: *δ* 47.24, 98.85, 127.06, 127.42, 128.52, 128.55, 129.08, 133.32, 137.45, 154.85, 178.37, 197.18. MS (EI, 70 eV): *m*/*z* (%) = 283 (5), 267(68), 181(7), 91 (100), 77 (4), 65 (12), 45 (4). Anal. Calcd. for C_16_H_13_NS_2_ (283): C, 67.81; H, 4.62; N, 4.94. Found: C, 67.70; H, 4.52; N, 4.73 %.

*3-(3,4-dichlorobenzyl)-4-phenyl-1,3-thiazole-2(3H)-thione* (**4b**): Colorless viscous oil; FT-IR (KBr): = 3152, 3004, 1628, 1603, 1477, 1302, 1104 cm^-1^. ^1^H NMR (250 MHz, CDCl_3_*)*: *δ* 4.83 (s, 2H, CH_2_*)*, 6.05 (CH of alkene), 6.75–7.97 (m, 8H, CH of ArH). ^13^C NMR (62.5 MHz, CDCl_3_*)*: *δ* 46.07, 99.25, 126.72, 128.65, 128.74, 129.35, 129.66, 133.54, 130.50, 135.38, 136.62, 137.21, 172.70, 194.15. Anal. Calcd. for C_16_H_11_Cl_2_NS_2_ (350): C, 54.55; H, 3.15; N, 3.98. Found: C, 54.36; H, 3.05; N, 3.84 %.

*3-(2-Naphthyl methyl)-4-phenyl-1,3-thiazole-2(3H)-thione* (**4c**): Colorless viscous oil; FT-IR (KBr): = 3102, 3009, 1652, 1605, 1479, 1204 cm^-1^. ^1^H NMR (250 MHz, CDCl_3_*)*: *δ* 3.95 (s, 2H, CH_2_*)*, 6.12 (s, 1H, CH of alkene), 6.92–7.97 (m, 12H, CH of ArH). ^13^C NMR (62.5 MHz, CDCl_3_*)*: *δ* 45.35, 99.05, 123.77, 125.32, 125.84, 126.34, 128.06, 128.68, 128.75, 133.54, 122.52, 129.28, 131.50, 135.08, 172.44, 194.16. Anal. Calcd. for C_20_H_15_NS_2_ (333): C, 72.03; H, 4.53; N, 4.20. Found: C, 72.05; H, 4.40; N, 4.15 %.

*3-(2-Furyl methyl)-4-phenyl-1,3-thiazole-2(3H)-thione* (**4d**): Colorless viscous oil; FT-IR (KBr): = 3105, 3002, 1653, 1607, 1474, 1202 cm^-1^. ^1^H NMR (250 MHz, CDCl_3_*)*: *δ* 4.84 (s, 2H, CH_2_*)*, 6.10 (s, 1H, CH of alkene), 6.22 (1H, CH of furan), 7.25–8.05 (m, 7H, CH of ArH and CH of furan). ^13^C NMR (62.5 MHz, CDCl_3_*)*: *δ* 44.25, 98.32, 109.52, 110.83, 127.08, 128.76, 129.58, 142.12, 144.54, 147.92, 155.44, 192.18. Anal. Calcd. for C_14_H_11_NOS_2_ (273): C, 61.51; H, 4.06; N, 5.12. Found: C, 61.46; H, 4.04; N, 5.09 %.

*3-(4-Fluorobenzyl)-4-phenyl-1,3-thiazole-2(3H)-thione* (**4e**):

Colorless viscous oil; FT-IR (KBr): = 3153, 3005, 1628, 1604, 1473, 1302, 1108 cm^-1^. ^1^H NMR (250 MHz, CDCl_3_*)*: *δ* 4.85 (s, 2H, CH2*)*, 6.05 (s, 1H, CH of alkene), 6.85 (d, 2H, *J* = 6.8 Hz, CH arom), 7.02–7.59 (m, 5H, CH of ArH), 7.98 (d, 2H, *J* = 7.5 Hz, CH of ArH).^13^C NMR (62.5 MHz, CDCl_3_*)*: *δ* 46.45, 99.08, 114.53, 128.67, 128.78, 133.51, 129.05, 135.46, 137.57, 153.28, 159.50, 194.19. Anal. Calcd. for C_16_H_12_FNS_2_ (301): C, 63.76; H, 4.01; N, 4.65. Found: C, 63.60; H, 4.04; N, 4.42 %.

*3-(2-Methoxybenzyl)-4-phenyl-1,3-thiazole-2(3H)-thione* (**4f**): Colorless viscous oil; FT-IR (KBr): = 3150, 3000, 1650, 1600, 1470, 1200, 1100 cm^-1^. ^1^H NMR (250 MHz, CDCl_3_*)*: *δ* 3.62 (s, 3H, OCH_3_*)*, 4.90 (s, 2H, CH_2_*)*, 6.03 (s, 1H, CH of alkene), 6.71–7.98 (m, 9H, CH, ArH).^13^C NMR (62.5 MHz, CDCl_3_*)*: *δ* 42.56, 55.05, 98.47, 110.05, 120.53, 128.38, 128.46, 128.55, 128.78, 129.05, 133.54, 127.12, 135.45, 156.35, 194.14. Anal. Calcd. for C_17_H_15_NOS_2_ (313): C, 65.14; H, 4.82; N, 4.47. Found: C, 65.03; H, 4.74; N, 4.35 %.

*3-(4-Methylbenzyl)-4-(4-methoxyphenyl)-1,3-thiazole-2(3H)-thione* (**4g**):

Colorless viscous oil; FT-IR (KBr): = 3156, 3008, 1648, 1612, 1475, 1206, 1108 cm^-1^. ^1^H NMR (250 MHz, CDCl_3_*)*: *δ* 2.23 (s, 3H, CH_3_), 3.86 (s, 3H, OCH_3_*)*, 4.95 (s, 2H, CH2*)*, 5.98 (s, 1H, CH of alkene), 6.82–7.35 (m, 8H, CH of ArH).^13^C NMR (62.5 MHz, CDCl_3_*)*: *δ* 21.35, 48.54, 55.95, 98.68, 115.38, 123.42, 125.64, 130.65, 131.25, 132.59, 139.25, 160.20, 174.25, 183.56. Anal. Calcd. for C_18_H_17_NOS_2_ (327): C, 66.02; H, 5.23; N, 4.28;. Found: C, 65.90; H, 5.14; N, 4.12 %.

*3-benzyl-4-(4-methoxyphenyl)-1,3-thiazole-2(3H)-thione* (**4h**)

Colorless viscous oil; FT-IR (KBr): = 3157, 3012, 1645, 1616, 1478, 1209, 1107 cm^-1^. ^1^H NMR (250 MHz, CDCl_3_*)*: *δ* 3.89 (s, 3H, OCH_3_*)*, 5.25 (s, 2H, CH_2_*)*, 6.28 (s, 1H, CH of alkene), 6.85–7.39 (m, 9H, CH of ArH).^13^C NMR (62.5 MHz, CDCl_3_*)*: *δ* 48.50, 55.37, 99.86, 110.55, 114.54, 122.54, 128.38, 129.54, 132.86, 137.54, 145.68, 160.85, 185.36. MS (EI, 70 eV): *m*/*z* (%) = 313 (M). Anal. Calcd. for C_17_H_15_NOS_2_ (313): C, 65.14; H, 4.82; N, 4.47; Found: C, 65.02; H, 4.56; N, 4.34; %.

*3-(2-Furyl methyl)-4-(4-methoxyphenyl)-1,3-thiazole-2(3H)-thione* (**4i**):

Colorless viscous oil; FT-IR (KBr): = 3144, 3012, 1658, 1615, 1478, 1209, 1112 cm^-1^. ^1^H NMR (250 MHz, CDCl_3_*)*: *δ* 3.88 (s, 3H, OCH3*)*, 4.82 (s, 2H, CH2*)*, 5.98 (2H, CH of furan), 6.20 (s, 1H, CH of alkene), 6.75–7.42 (m, 5H, CH of furan and CH of ArH). ^13^C NMR (62.5 MHz, CDCl_3_*)*: *δ* 41.35, 55.34, 98.36, 108.35, 110.35, 118.35, 122.54, 130.22, 138.54, 142.35, 150.65, 161.25, 178.25. Anal. Calcd. for C_15_H_13_NO_2_S_2_ (303): C, 59.38; H, 4.32; N, 4.62; Found: C, 59.15; H, 4.14; N, 4.42. %.

*3-(2-methoxybenzyl)-4-(4-methoxyphenyl)-1,3-thiazole-2(3H)-thione* (**4j**):

Colorless viscous oil; FT-IR (KBr): = 3142, 3010, 1654, 1611, 1472, 1205, 1116 cm^-1^. ^1^H NMR (250 MHz, CDCl_3_*)*: *δ* 3.68 (s, 3H, OCH_3_), 3.84 (s, 3H, OCH_3_*)*, 4.89 (s, 2H, CH_2_*)*, 6.05 (s, 1H, CH of alkene), 6.72–7.53 (m, 8H, CH of ArH). ^13^C NMR (62.5 MHz, CDCl_3_*)*: *δ* 43.54, 56.45, 56.48, 98.45, 110.25, 115.28, 120.54, 122.54, 125.85, 125.64, 128.54, 130.42, 138.20, 158.64, 160.24, 172.54. Anal. Calcd. for C_18_H_17_NO_2_S_2_ (343): C, 62.94; H, 4.99; N, 4.08; Found: C, 62.72; H, 4.70; N, 3.91. %.

FT-IR: 3-Benzyl-4-phenyl-1,3-thiazole-2(3H)-thione (**4a**):


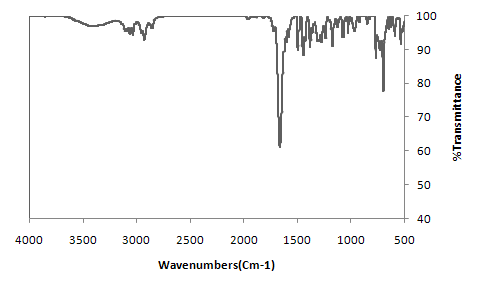


^1^H NMR: 3-Benzyl-4-phenyl-1,3-thiazole-2(3H)-thione (**4a**):


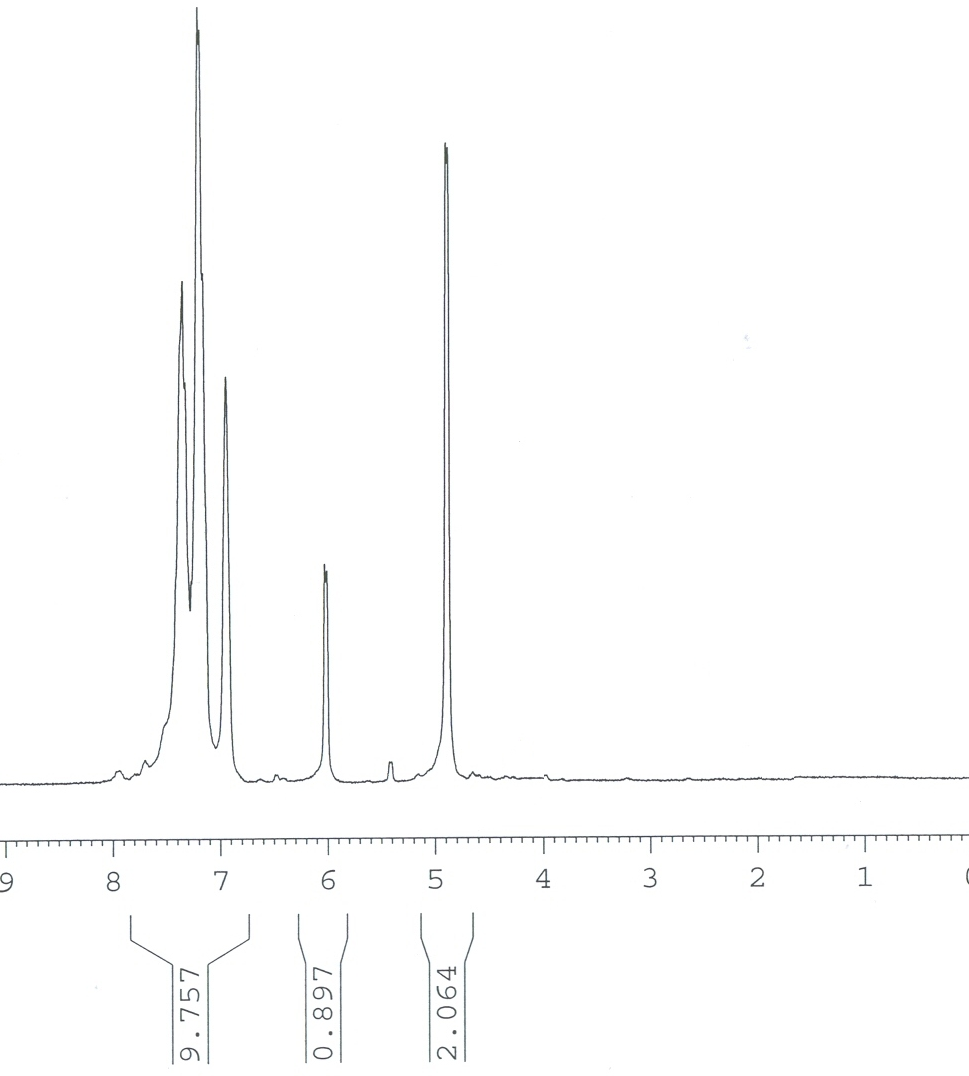


^13^C NMR: 3-Benzyl-4-phenyl-1,3-thiazole-2(3H)-thione (**4a**):


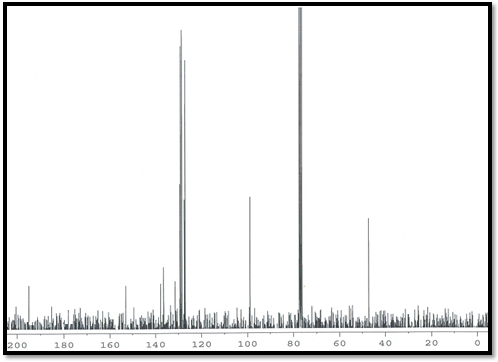


FT-IR: 3-(3,4-dichlorobenzyl)-4-phenyl-1,3-thiazole-2(3H)-thione (**4b**):


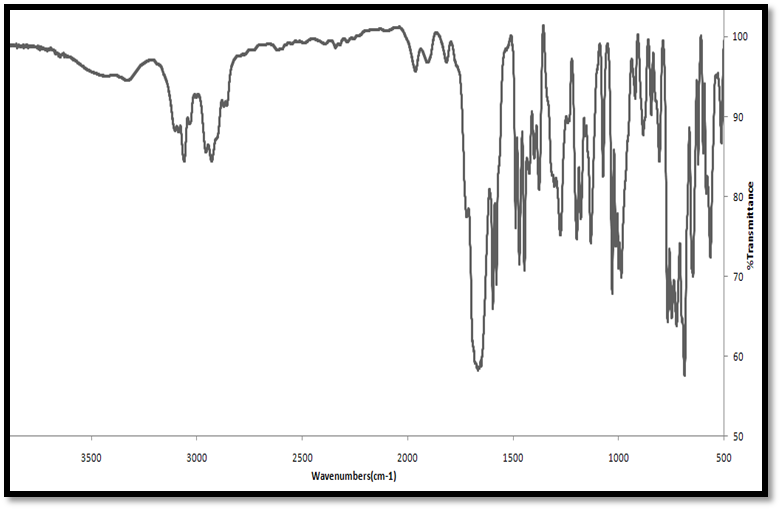


^1^H NMR: 3-(3,4-dichlorobenzyl)-4-phenyl-1,3-thiazole-2(3H)-thione (**4b**):


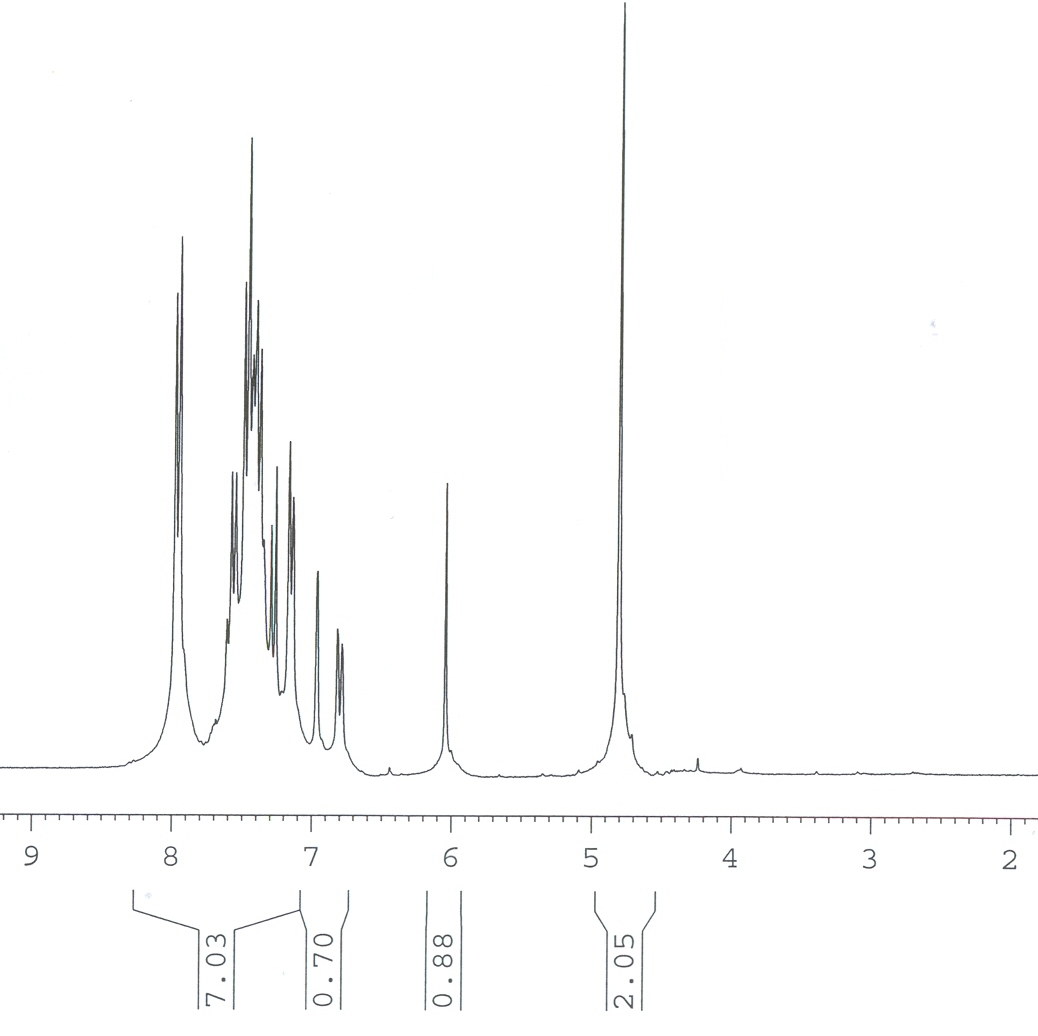


^13^C NMR: 3-(3,4-dichlorobenzyl)-4-phenyl-1,3-thiazole-2(3H)-thione (**4b**):


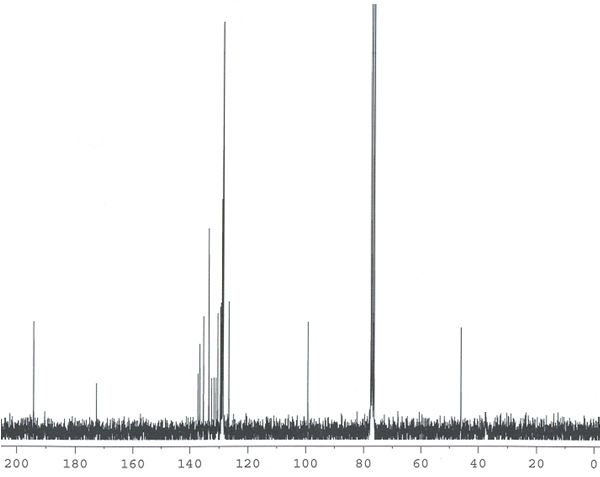


FT-IR: 3-(2-Naphthyl methyl)-4-phenyl-1,3-thiazole-2(3H)-thione (**4c**):


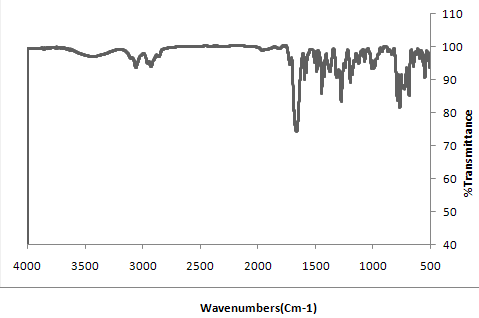


^1^H NMR: 3-(2-Naphthyl methyl)-4-phenyl-1,3-thiazole-2(3H)-thione (**4c**):


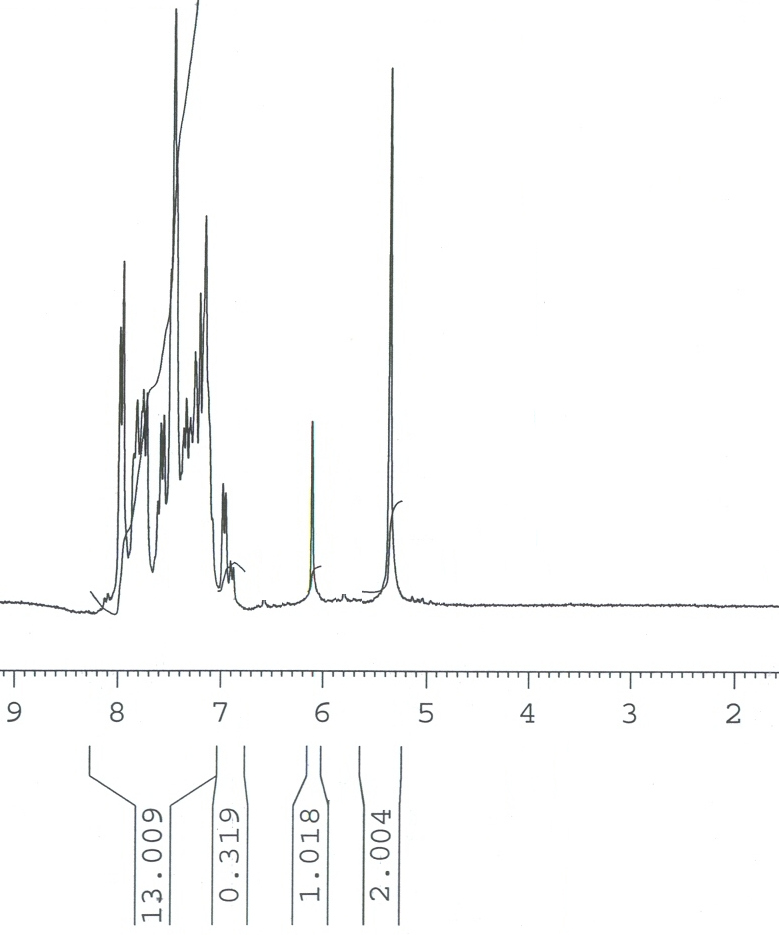


^13^C NMR: 3-(2-Naphthyl methyl)-4-phenyl-1,3-thiazole-2(3H)-thione (**4c**):


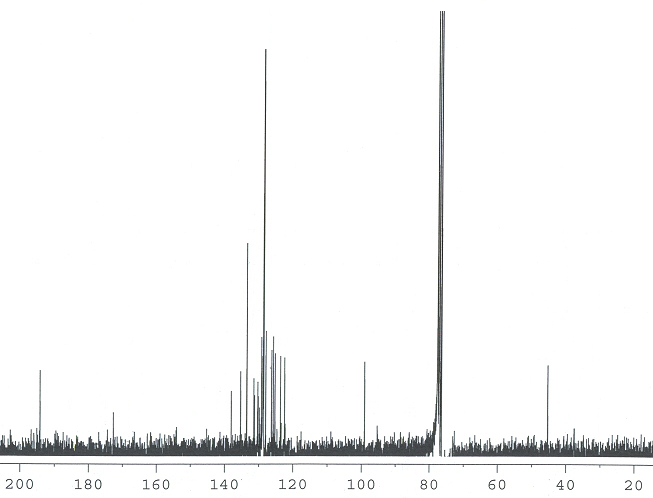


FT-IR: 3-(2-Furyl methyl)-4-phenyl-1,3-thiazole-2(3H)-thione (**4d**):


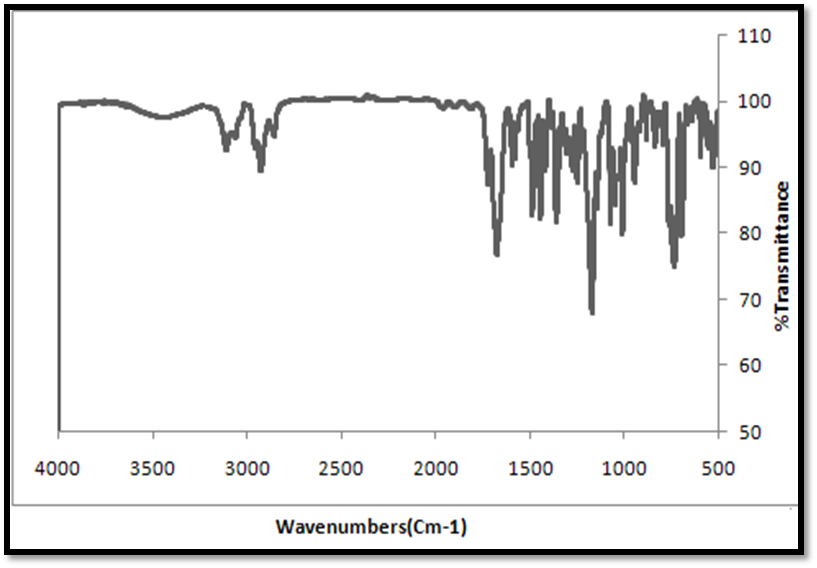


^1^H NMR: 3-(2-Furyl methyl)-4-phenyl-1,3-thiazole-2(3H)-thione (**4d**):


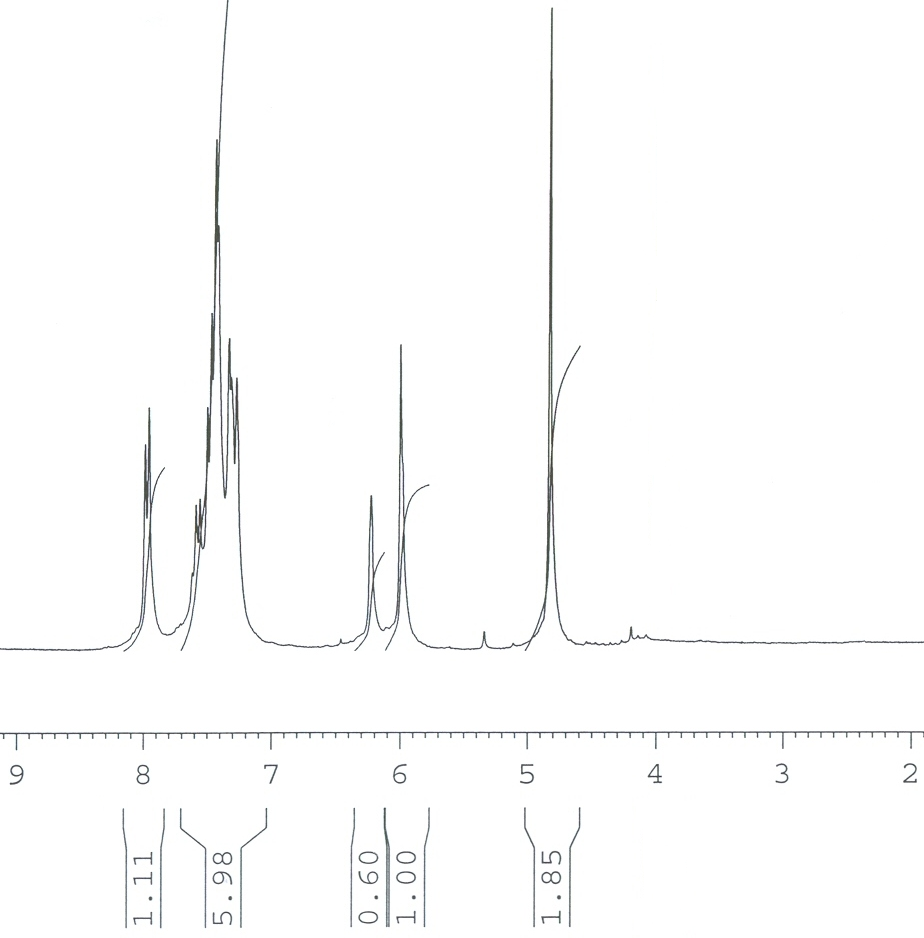


^13^C NMR: 3-(2-Furyl methyl)-4-phenyl-1,3-thiazole-2(3H)-thione (**4d**):


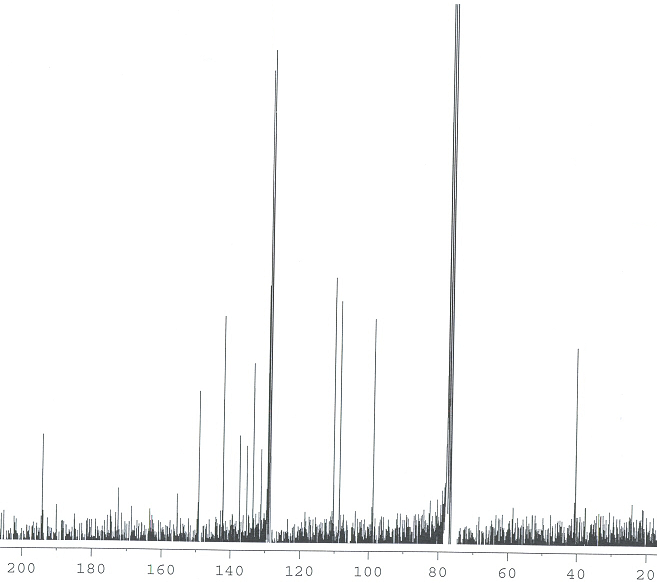


FT-IR: 3-(4-Fluorobenzyl)-4-phenyl-1,3-thiazole-2(3H)-thione (**4e**):


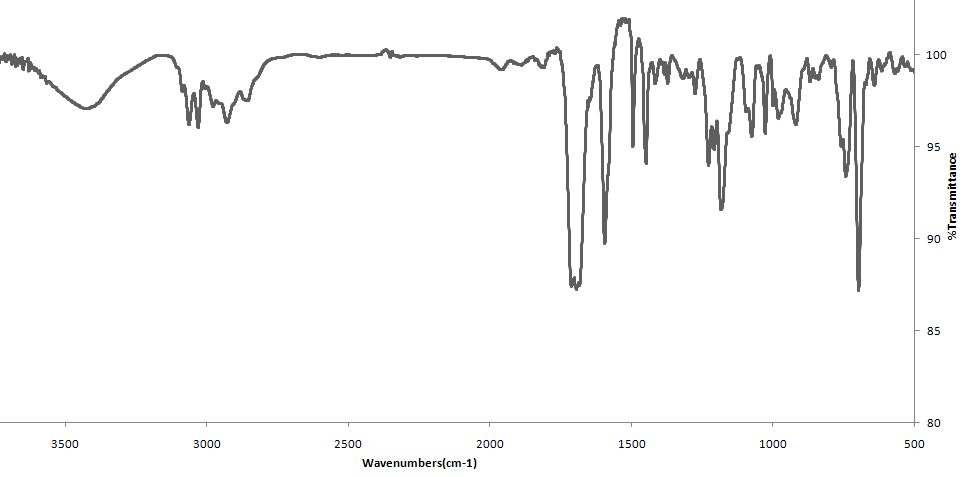


^1^H NMR: 3-(4-Fluorobenzyl)-4-phenyl-1,3-thiazole-2(3H)-thione (**4e**):


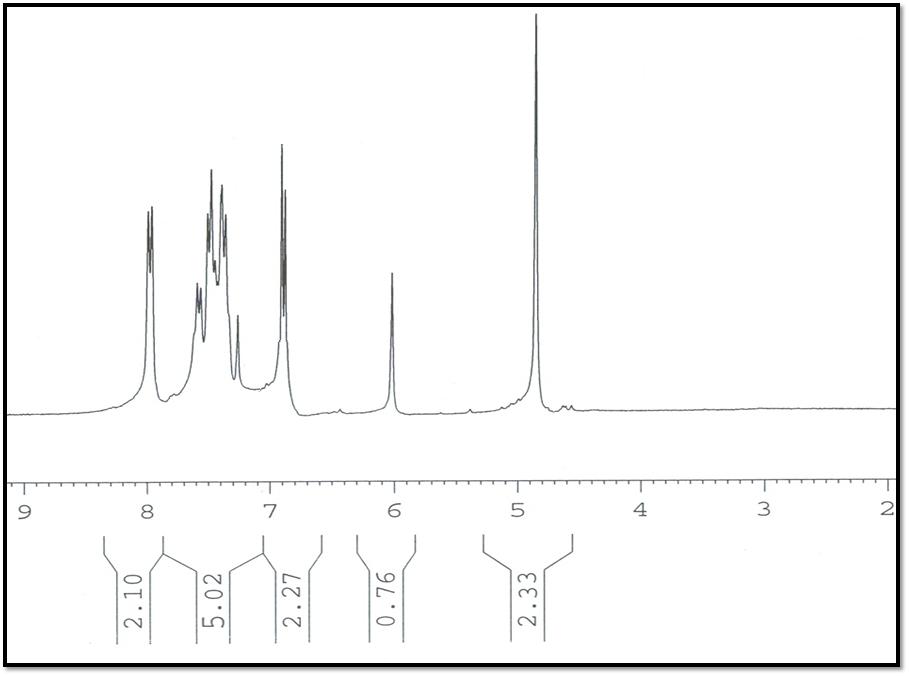


^13^C NMR: 3-(4-Fluorobenzyl)-4-phenyl-1,3-thiazole-2(3H)-thione (**4e**):


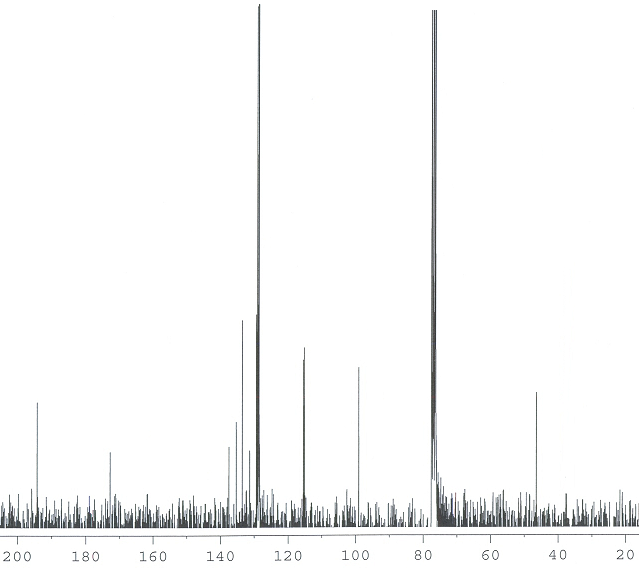


FT-IR 3-(2-Methoxybenzyl)-4-phenyl-1,3-thiazole-2(3H)-thione (**4f**):


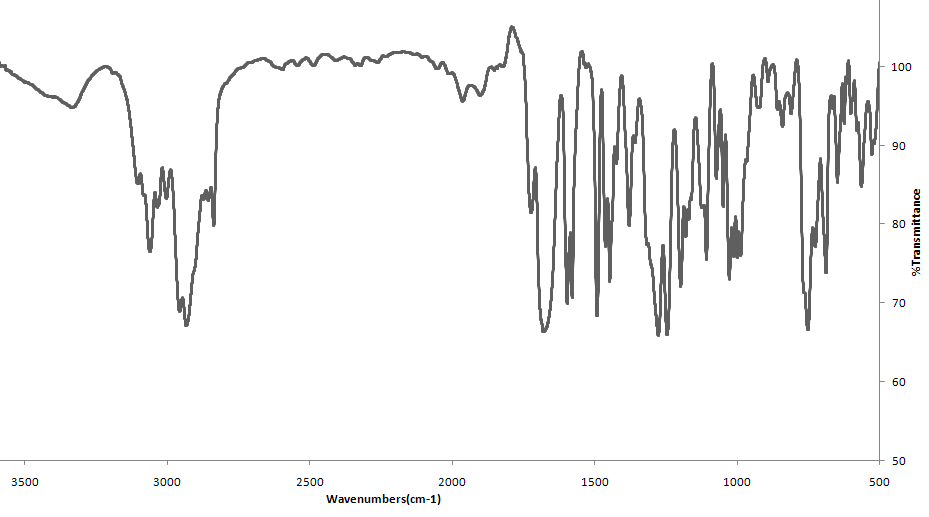


^1^H NMR 3-(2-Methoxybenzyl)-4-phenyl-1,3-thiazole-2(3H)-thione (**4f**):


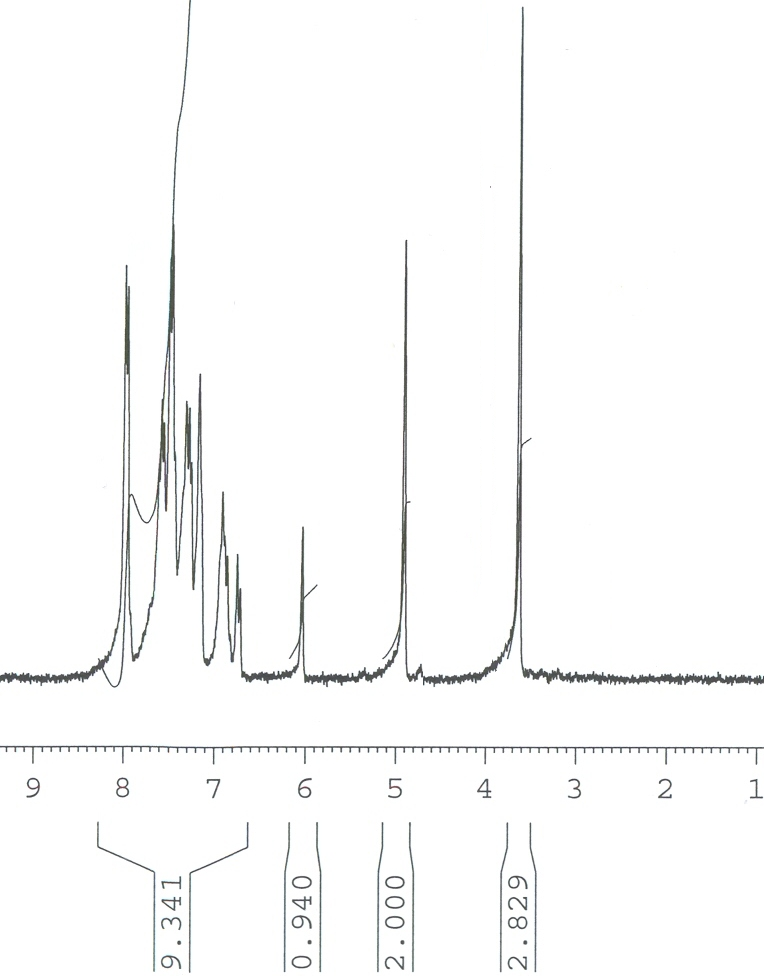


FT-IR 3-(4-Methylbenzyl)-4-(4-methoxyphenyl)-1,3-thiazole-2(3H)-thione (**4g**):


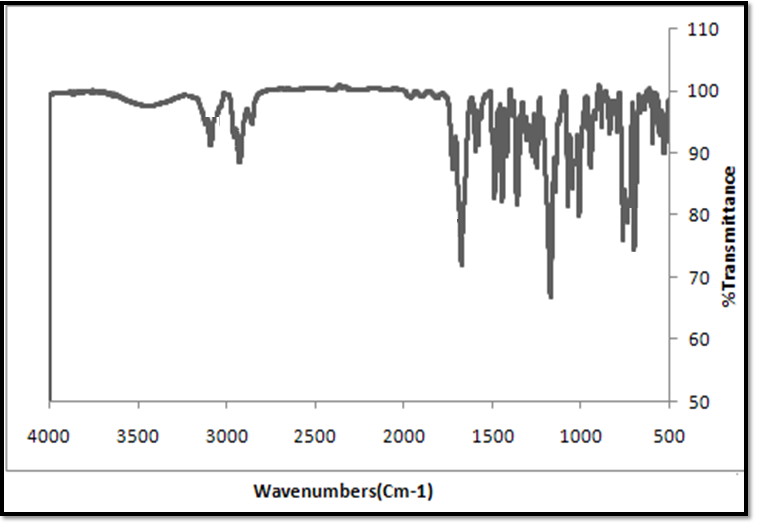


^1^H NMR 3-(4-Methylbenzyl)-4-(4-methoxyphenyl)-1,3-thiazole-2(3H)-thione (**4g**):


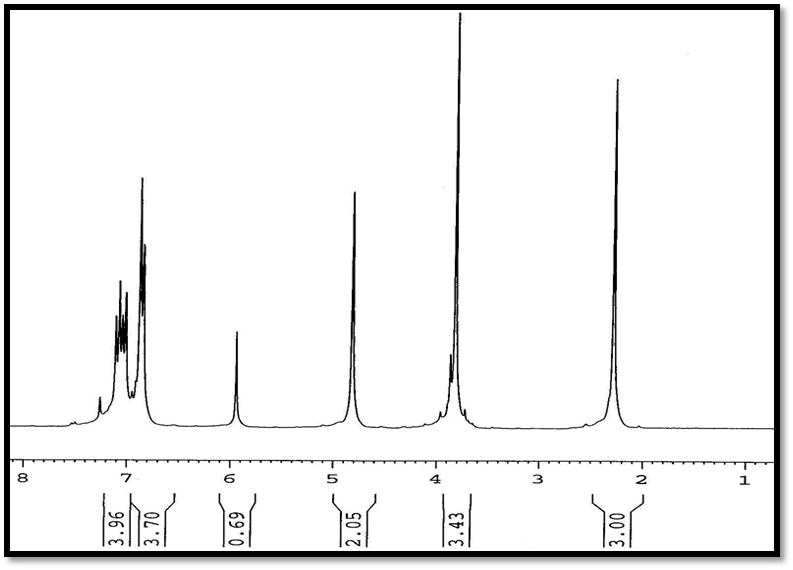


^13^C NMR 3-(4-Methylbenzyl)-4-(4-methoxyphenyl)-1,3-thiazole-2(3H)-thione (**4g**):


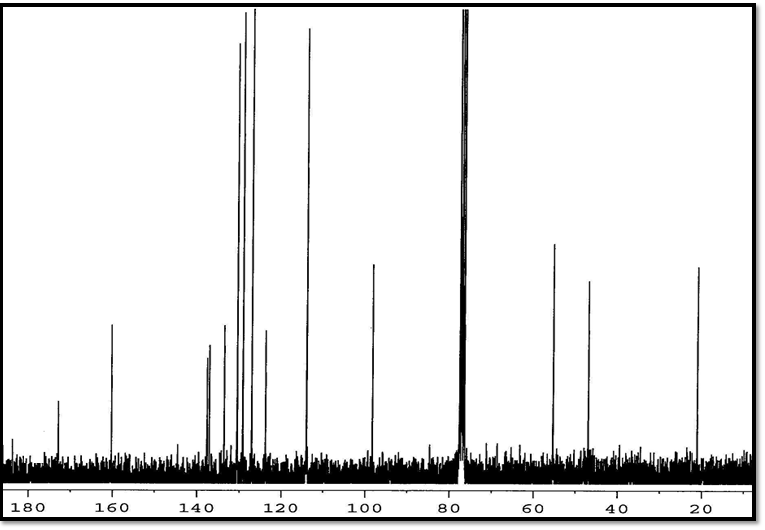


FT-IR: 3-benzyl-4-(4-methoxyphenyl)-1,3-thiazole-2(3H)-thione (**4h**)


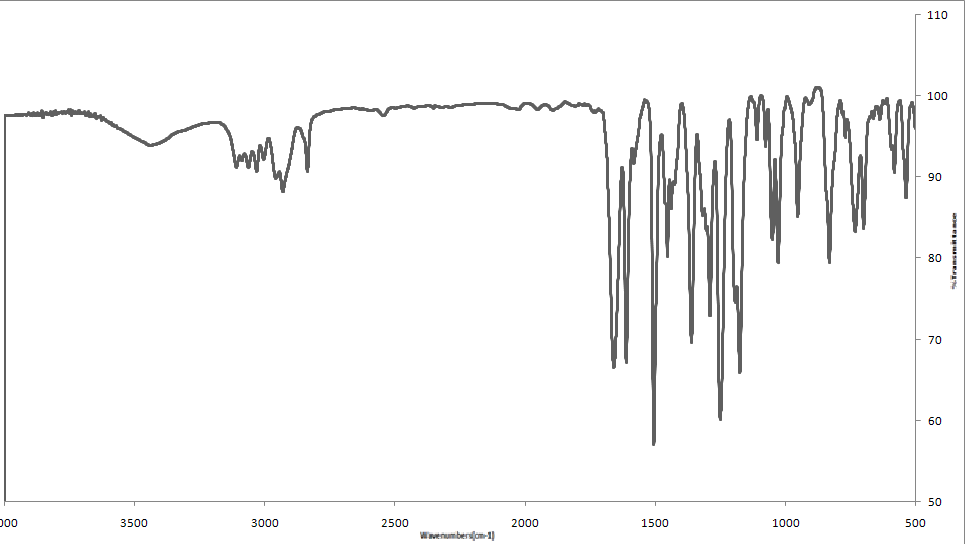


^1^H NMR 3-benzyl-4-(4-methoxyphenyl)-1,3-thiazole-2(3H)-thione (**4h**)


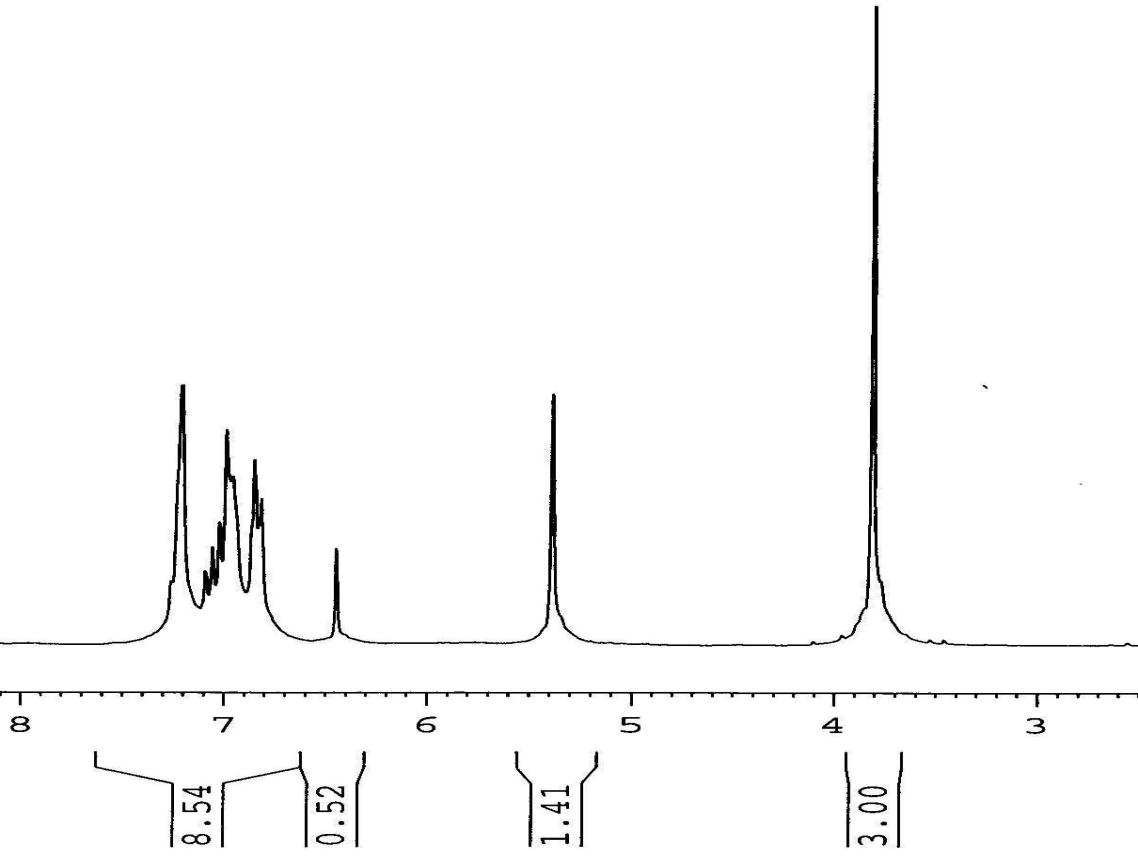


^13^C NMR 3-benzyl-4-(4-methoxyphenyl)-1,3-thiazole-2(3H)-thione (**4h**)


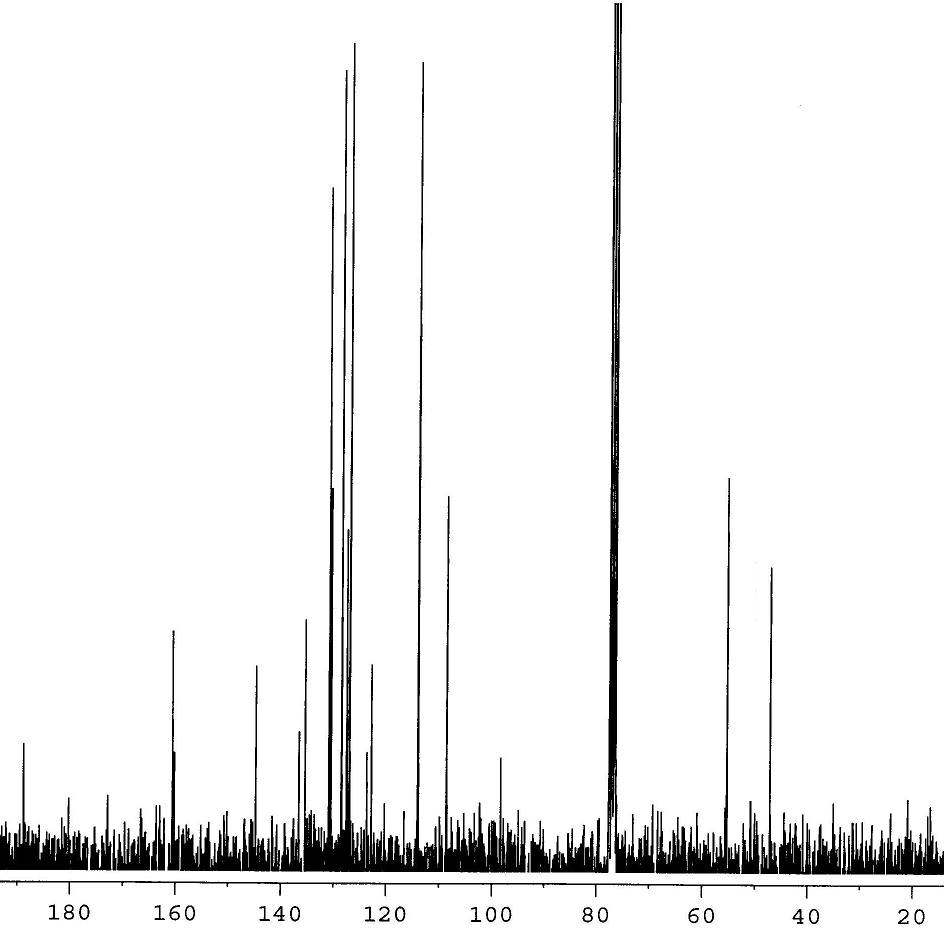


Mass spectrum (MS): 3-benzyl-4-(4-methoxyphenyl)-1,3-thiazole-2(3H)-thione (**4h**)


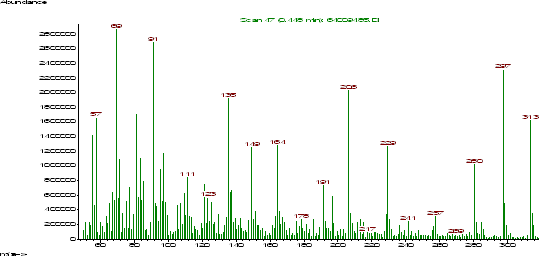


FT-IR 3-(2-Furyl methyl)-4-(4-methoxyphenyl)-1,3-thiazole-2(3H)-thione (**4i**):


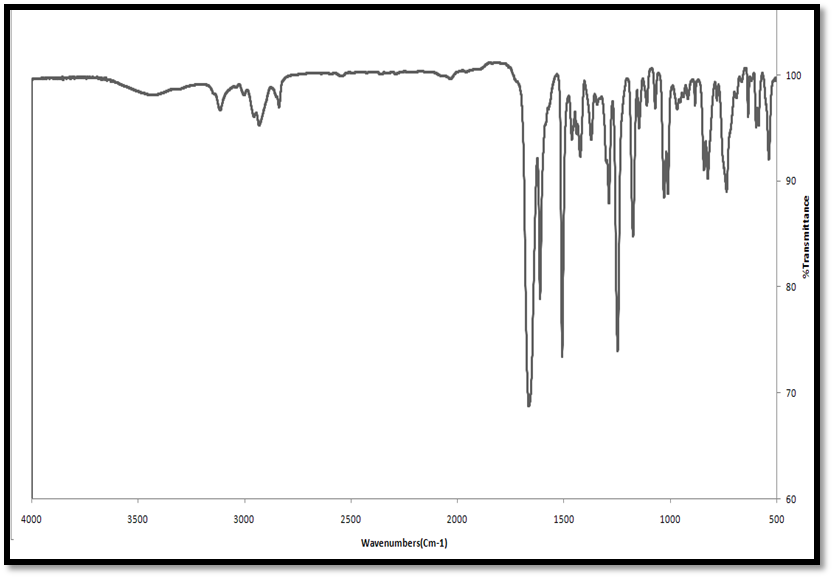


^1^H NMR 3-(2-Furyl methyl)-4-(4-methoxyphenyl)-1,3-thiazole-2(3H)-thione (**4i**):


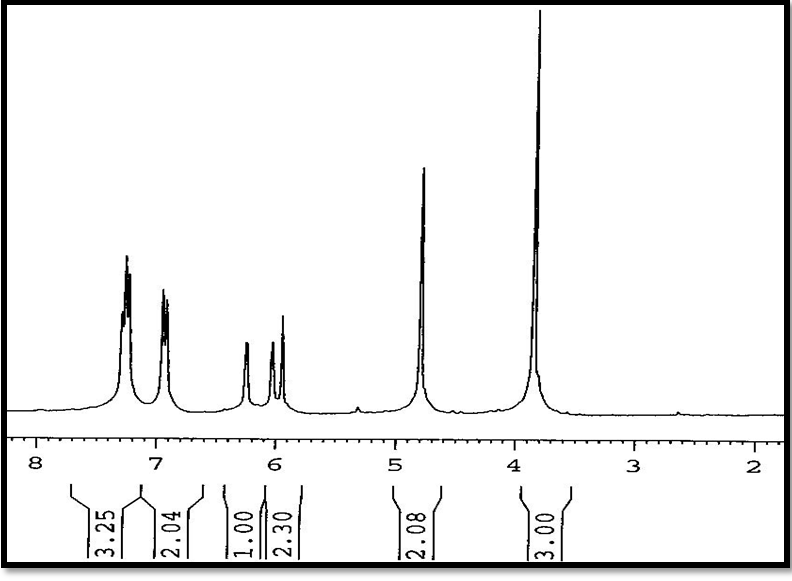


^13^C NMR 3-(2-Furyl methyl)-4-(4-methoxyphenyl)-1,3-thiazole-2(3H)-thione (**4i**):


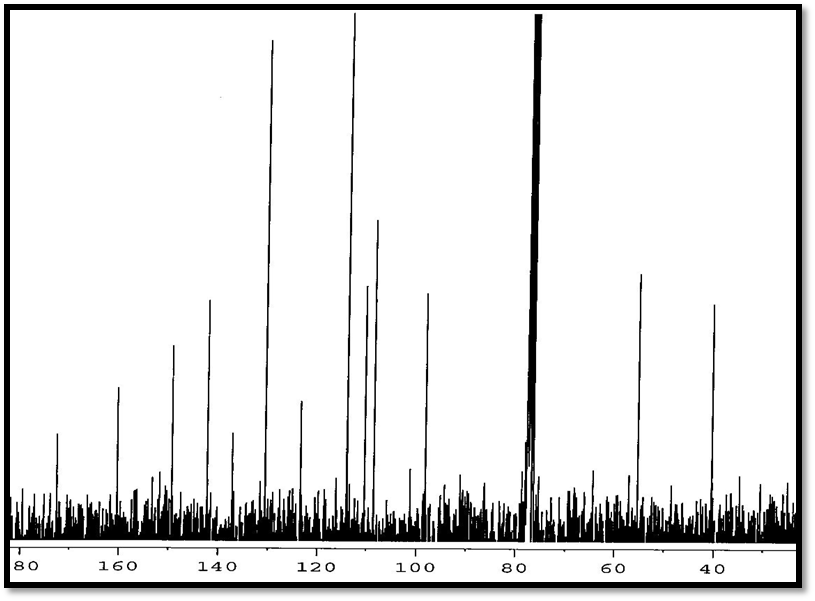


FT-IR 3-(2-Methoxybenzyl)-4-(4-methoxyphenyl)-1,3-thiazole-2(3H)-thione (**4j**):


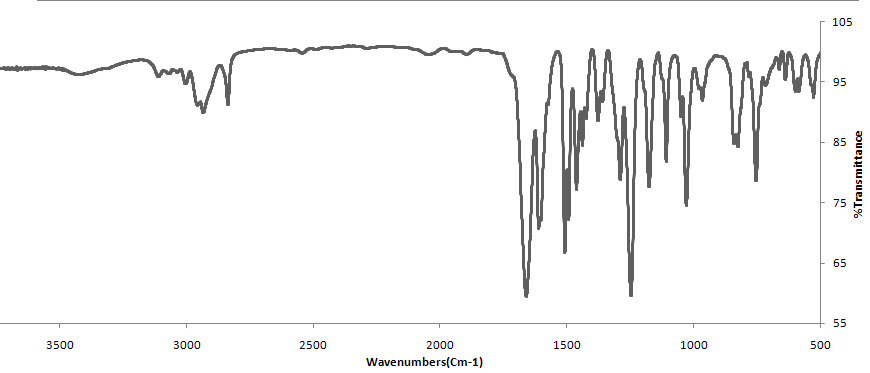


^1^H NMR 3-(2-Methoxybenzyl)-4-(4-methoxyphenyl)-1,3-thiazole-2(3H)-thione (**4j**):


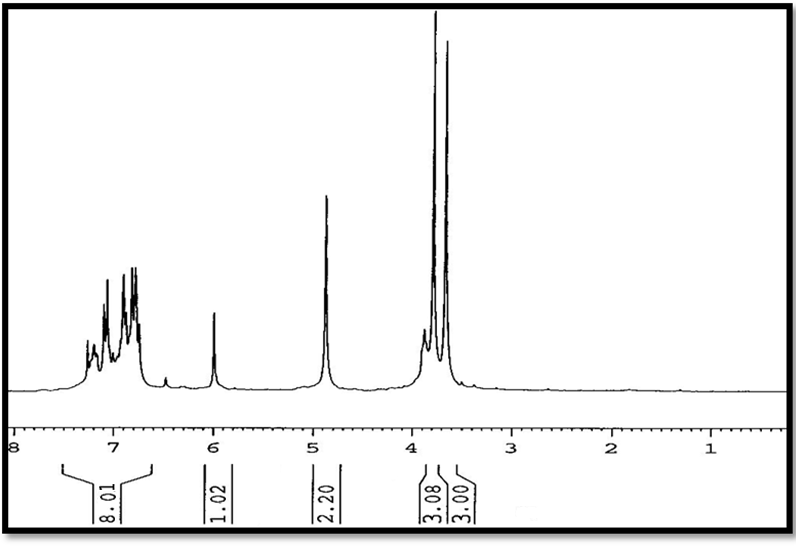


^13^ C NMR 3-(2-Methoxybenzyl)-4-(4-methoxyphenyl)-1,3-thiazole-2(3H)-thione (**4j**):


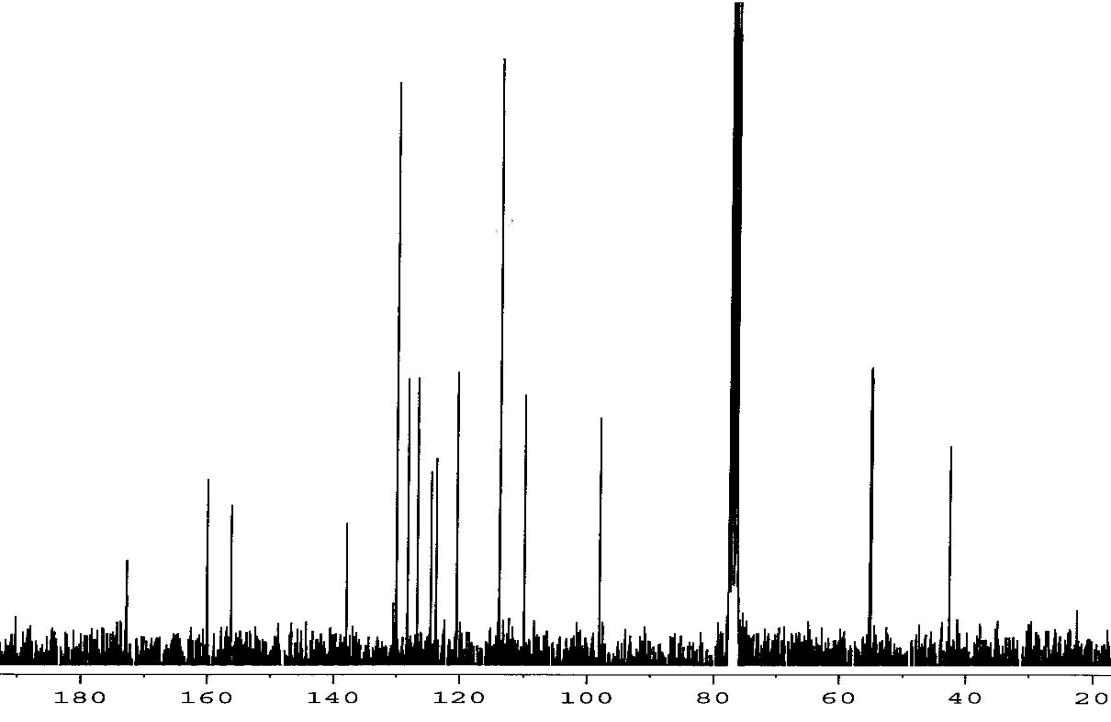

Supplement: Supplementary file 1 — Additional file 1. The spectral data of products are described in the additional file 1. [file 13065_2019_637_MOESM1_ESM.docx]
